# UV light-emitting diode (UV-LED) at 265 nm as a potential light source for disinfecting human platelet concentrates

**DOI:** 10.1371/journal.pone.0251650

**Published:** 2021-05-20

**Authors:** Tomoya Hayashi, Kumiko Oguma, Yoshihiro Fujimura, Rika A. Furuta, Mitsunobu Tanaka, Mikako Masaki, Yasuhito Shinbata, Takafumi Kimura, Yoshihiko Tani, Fumiya Hirayama, Yoshihiro Takihara, Koki Takahashi

**Affiliations:** 1 Japanese Red Cross Kinki Block Blood Centre, Ibaraki, Osaka, Japan; 2 The University of Tokyo, Tokyo, Japan; 3 Central Blood Institute, Japanese Red Cross, Tokyo, Japan; 4 Blood Service Headquarters, Japanese Red Cross, Tokyo, Japan; Massachusetts General Hospital, UNITED STATES

## Abstract

The risk of sepsis through bacterial transmission is one of the most serious problems in platelet transfusion. In processing platelet concentrates (PCs), several methods have been put into practice to minimize the risk of bacterial transmission, such as stringent monitoring by cultivation assays and inactivation treatment by photoirradiation with or without chemical agents. As another potential option, we applied a light-emitting diode (LED) with a peak emission wavelength of 265 nm, which has been shown to be effective for water, to disinfect PCs. In a bench-scale UV-LED exposure setup, a 10-min irradiation, corresponding to an average fluence of 9.2 mJ/cm^2^, resulted in >2.0 log, 1.0 log, and 0.6 log inactivation (mean, n = 6) of *Escherichia coli*, *Staphylococcus aureus*, and *Bacillus cereus*, respectively, in non-diluted plasma PCs. After a 30-min exposure, platelet counts decreased slightly (18 ± 7%: mean ± SD, n = 7); however, platelet surface expressions of CD42b, CD61, CD62P, and PAC-1 binding did not change significantly (P>0.005), and agonist-induced aggregation and adhesion/aggregation under flow conditions were well maintained. Our findings indicated that the 265 nm UV-LED has high potential as a novel disinfection method to ensure the microbial safety of platelet transfusion.

## Introduction

Platelets play a central role in stopping bleeding under either low or high flow rate conditions through surface molecules including glycoproteins, such as GPIb/IX/V (CD42) and GPIIb/IIIa (CD41/CD61), and released molecules in platelet granules, which activate the surrounding platelets. Platelet concentrates (PCs) are commonly transfused to stop bleeding in thrombocytopenia patients who have active bleeding and are also used prophylactically for patients receiving chemotherapy [1–3]. PCs are usually stored at room temperature (20–24°C) with continuous agitation; however, this storage condition allows any contaminating bacteria to proliferate quickly. A previous report by the Japanese Red Cross showed that positive BacT/ALERT in outdated PCs, which were obtained more than 4 days after collection, occurred at a frequency of 0.05% (11 out of 21,783 PCs), and the number of contaminations of clinical importance was 4 of 21,786 PCs (0.018%) excluding those positive for *Propionibacterium acnes* [4]. Cold storage of platelets has been studied and developed since the 1950s and can prolong storage duration [5]. However, cold-stored platelets have markedly reduced *in vivo* survival after transfusion, despite enhanced platelet aggregability due to their activation under low temperatures, and room temperature storage consistently shows longer *in vivo* platelet circulation times [6, 7]. Hence, cold-storage platelets are preferentially used for haemostasis in surgical interventions, and platelets kept at room temperature are routinely used. Thus, the shelf-life of PC products is relatively short (3 to 5 days) in countries without a bacterial screening system or pathogen inactivation system [8]. Unfortunately, sepsis after transfusion of bacterially contaminated PCs, irrespective of shelf-life, cannot be fully prevented [9]. To mitigate the risk of bacterial contamination in PCs, two major strategies have been introduced in many advanced countries during the past two decades: culture-based detection of bacteria and pathogen inactivation procedures [8, 10, 11].

Murphy et al. [12] found that culture-based detection of bacteria is highly sensitive, but is not perfect because bacteria grow during the storage of PCs even with a low count (less than 1 colony-forming unit (cfu)/mL) from contamination at early storage; the detection rate of bacterial contamination increased from 14.9% for sampling on day 1 to 100% for sampling on day 7 [11, 13]. In the UK, culture-based detection of bacteria undertaken with a combination large volume sampling (15 mL) and delayed sampling (at 36 to 48 h after PC collection) has been introduced since 2011 with excellent results [14], and thereafter blood manufacturers and suppliers in some countries have followed the UK system. In addition, the United States Food and Drug Administration (FDA) now encourages one of the following three options [15]: (1) the same method as the UK system described above as a single-step strategy, though the expiration day differs in sampling point after collection; (2) a two-step method for culture-based detection of bacteria, in which the 1st test is performed after PC collection and the 2nd test is carried out at four days after PC collection, resulting in the extension of clinical use for a maximum of seven days, though at the expense of less product volume; or (3) the introduction of a bacterial sterilization process that enables clinical use for five days after collection without a bacterial culture test.

For pathogen inactivation, the Intercept® (Cerus Corporation, Concord, CA) and Mirasol® (Terumo BCT, Lakewood, CO) systems are currently commercially available in many countries [16, 17]. They are designed for either ultraviolet (UV) A irradiation with psoralen or UV light (280–400 nm) irradiation with riboflavin (vitamin B_2_), both of which have shown high efficacy to reduce a variety of pathogens [17]. More recently, Theraflex® (MacoPharma, Mouvaux, France) has been developed as a third option, and uses short wavelength UVC (254 nm) without any added photosensitizer. The successful application of this approach has been highly anticipated because of its simple procedure, and a phase III clinical trial is currently under way [18]. In general, however, platelets tend to show greater functional damage following exposure to UVC than UVA or UVB, since radiation with a shorter wavelength possesses greater energy [19]. Despite a comparable principle of photochemical inactivation, all three methods affect platelets in different ways [20]. Intercept may result in more apoptosis than in untreated platelets; Mirasol occasionally leads to unacceptable swirling, and 10% of product must be discarded due in part to the lowered pH of 6.4; Theraflex-treated platelets are associated with increased lactic acid production during storage and the enhanced exposure of CD62P and phosphatidylserine on platelet surfaces compared to their untreated counterparts. The main properties of these three methods are summarized in Table 1.

**Table 1:** Characteristics of pathogen inactivation systems [17, 20, 21].

Technique and equipment	Intercept	Mirasol	Theraflex
UV light	UVA	Broad spectrum	UVC
	(320–400 nm)	(280–400 nm)	(254 nm)
Photosensitizer	Amotosalen	Riboflavin	None
Target damage	Intercalation in helical regions	Oxido-reductive damage	Pyrimidine dimerization
(how it prevents DNA proliferation)			
Typical consequences	Amotosalen remains on platelets due to lipid binding	Increases platelet anaerobic metabolism rates	“Primes” platelets for activation due to reduced disulphide bonds
Availability	Commercial	Commercial	Not yet in routine use

Light-emitting diodes (LEDs) with germicidal UV emission, hereafter referred to as UV-LEDs, are an emerging source of UV radiation, and they have been shown to be effective for water disinfection (for a review see [22]). The inactivation efficiencies of UV-LEDs with peak emission wavelengths of 265, 280, and 300 nm have been compared using various microorganisms suspended in phosphate buffer solution; 265 and 280 nm UV-LEDs are feasible options, with the 265 nm UV-LED showing the best fluence-based inactivation efficacy [23, 24]. However, neither studies on 265 nm UV-LED under a plasma milieu nor with PCs have been performed, because the low UV transmittance or high UV absorbance in both preparations represent a significant challenge. Proteins in blood plasma show a relative peak in absorbance at 280 nm, mainly due to the presence of the aromatic amino acids tryptophan and tyrosine; thus, emission at 265 nm rather than 280 nm would be more appropriate for the disinfection of PCs in plasma, thereby limiting protein damage.

In this context, the objective of this study was to evaluate the application of UV-LED at 265 nm as a novel disinfection method for platelet transfusion safety. We assessed the inactivation efficacy of UV-LED at 265 nm in three bacterial species, *Escherichia coli*, *Staphylococcus aureus*, and *Bacillus cereus*, suspended in human PCs. Furthermore, the degree of platelet activation and aggregation after UV-LED irradiation were tested to evaluate the potential negative impacts of UV-LED exposure on platelets.

## Materials and methods

All methods were performed in accordance with the Ethical Guidelines for Medical and Health Research Involving Human Subjects enacted by the Ministry of Health, Labour, and Welfare of Japan. The materials purchased for this study include: (1) phycoerythrin (PE)-conjugated anti-human CD62P (clone: AC1.2, mouse IgG), PE-conjugated mouse IgG (clone: MOPC-21), and peridinin chlorophyll protein complex (PerCP)-conjugated anti-human CD61 (clone: RUU-PL 7F12, mouse IgG) (BD Biosciences, Tokyo); (2) PE-conjugated anti-human CD42b antibody (clone: AN51; Agilent, Santa Clara, CA); (3) thrombin receptor-activating peptide (TRAP) (protease-activated receptor-1 agonist; Abcam, Tokyo); (4) adenosine 5’-diphosphate (ADP) (Sigma Aldrich Japan, Tokyo); (5) collagen reagent (Horm collagen; Moriya Sangyo, Tokyo); (6) *Escherichia coli* (DH-5α; F-, Φ80dlacZΔM15, Δ(lacZYA-argF) U169, deoR, recA1, endA1, hsdR17 (rK-, mK+), phoA, supE44, λ-, thi-1, gyrA96, relA1) (Takara Bio, Shiga, Japan); and (7) *Staphylococcus aureus* (NBRC 3060) and *Bacillus cereus* (NBRC 3001), gifted from the NITE Biological Resource Centre (Tokyo).

### Preparation and storage of platelet concentrates

After approval from the Ethics Committee of the Japanese Red Cross Blood Institute (2018–007), platelet concentrates (PCs) and blood samples were collected from healthy volunteers using aphaeresis instruments, Terusis-S (Terumo BCT Japan, Tokyo) or CCS (Haemonetics Japan Corporation, Tokyo), both of which are routinely employed in Japanese Red Cross Blood Centres. Written informed consent was obtained from all volunteers prior to sample collection. The harvested PCs contained more than 2 × 10^11^ platelets per bag and were irradiated with 15 Gy X-rays. The PCs were stored at 22°C with agitation before use within four days.

### Bacterial inoculation to PCs

One colony cultured overnight on a Luria-Bertani and soybean-casein-digested (LB-SCD) agar plate was picked and suspended in normal saline solution (Otsuka Pharmaceutical, Tokushima, Japan). The absorbance (550 nm) of the suspension was measured, and the suspension was then diluted with normal saline to a predetermined bacterial concentration. The strain of *E*. *coli* used in this study did not proliferate to form colonies efficiently when the bacteria were directly spiked to platelet concentrates. This inhibitory effect was calcium dependent and assumed to be via complement activation in sera, which can be totally blocked by ethylenediaminetetraacetic acid (EDTA) (see S1 Fig). Thus, we added EDTA in the assays using *E*. *coli* according to the methods described in two previous reports [25, 26]. An aliquot of the *E*. *coli* suspension was spiked to 10 mL of PC at final concentrations of 2 × 10^3^–8 × 10^3^ cfu/mL with EDTA (final concentration of 5 mmol/L) to negate the deleterious effects of plasma factors. The other two species, *S*. *aureus* and *B*. *cereus*, were also spiked into PCs, but without EDTA. Aliquots (100 μL) of the inoculated PCs were obtained at predetermined time-points (5, 10, 15, 20, 25, and 30 min) and incubated on LB-SCD agar for *E*. *coli* and the standard method agar DAIGO (Wako Pure Chemical, Osaka) for the other two species at 37°C for at least 12 h to determine cfu.

### UV-LED irradiation

**Fig 1:**
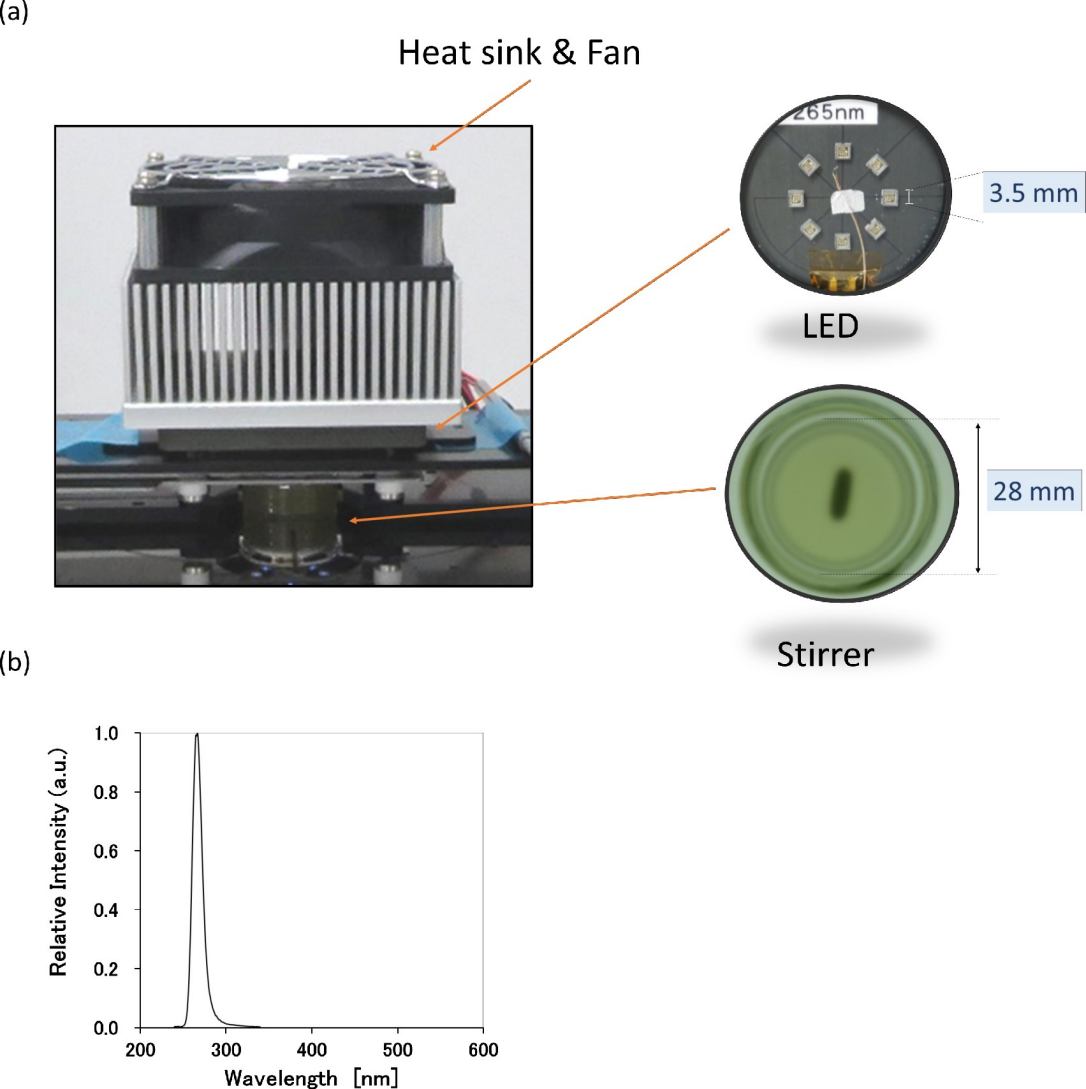
UV-LED irradiation setup. (a) Samples were placed in a siliconized quartz glass dish (28-mm inner diameter) with a stir bar. Eight UV-LED units (265 nm) were placed on the board face down for irradiation; the distance between the UV-LED and the sample surface before sampling was 17.7 mm. (b) The emission spectrum of the UV-LED used in this study.

All UV-LED irradiation was performed in a quartz glass petri dish (inner ϕ 28 mm × 15 mm; As One, Osaka). Approximately 2,750 μL of the PC-bacteria mixture was placed in the dish to obtain a sample depth of 5 mm. The dish was then irradiated with a bench-scale UV-LED setup, consisting of a circuit board with 8 UV-LED units (each LED unit was a 3.5 mm square with a peak emission wavelength of 265 nm (Nikkiso Giken, Tokyo)), a heat sink, and a cooling fan (Fig 1A and 1B). The UV-LEDs were operated at a constant direct current of 51.5 mA per unit, generating an incident irradiance of 1.05 mW/cm^2^ at the surface of the PC-bacteria mixture, as determined by ferrioxalate actinometry [27]. A 100-μL sample was obtained from one dish after each exposure: three or six samplings in total every 10 or 5 min were carried out; thus, the distance between the UV-LED and the sample surface increased in a stepwise manner with each sampling event, resulting in a slight stepwise reduction in incident irradiance up to 0.94 mW/cm^2^ after the fifth sampling event. The incident irradiance was corrected using the Beer-Lambert law, as described by Bolton and Linden [28], to correct for attenuation of UV light associated with photon absorption by the sample, and to determine the average irradiance delivered to the sample in the dish. The stepwise decrease in sample depth following each sampling event was also taken into account in calculating the average irradiance as determined by the Beer-Lambert law. The average fluence was determined as a product of the average irradiance and exposure time. During exposure, the samples were continuously stirred at 127 rpm by a magnetic stir bar coated with polytetrafluoroethylene at room temperature. At predetermined exposure times, an aliquot (ca. 100 μL) of the sample was removed and used for the following measurements (partly simultaneous measurements): (1) 50 μL, platelet counts using a Sysmex cell counter (Sx-800; Tokyo); (2) 2 μL, analysis of CD42b and CD61 surface expression and PAC-1 binding (below); (3) 50 μL, fixation with 1% paraformaldehyde (PFA) at 4°C overnight for microscopic analysis using a CKS41 microscope (Olympus, Tokyo) and CD62P expression analysis (below); (4) about 60 μL, diluted with AB plasma for agonist-induced platelet aggregation (below); and (5) 30 μL, diluted with AB plasma for platelet adherence and aggregation under a flow condition (below). The UV-LED irradiation experiment was independently conducted 30 times using different PC products.

### Surface expression of CD42b

Surface expression analysis was carried out according to the manufacturer’s instructions (BD Biosciences). In brief, the samples were incubated with both PerCP-conjugated anti-CD61 and PE-conjugated anti-human CD42b antibodies for 40 min at room temperature in the dark. After incubation, the samples were fixed with 1% PFA at 4°C overnight, and then washed with phosphate-buffered saline (PBS, pH 7.3). The treated samples were then applied to a SA3800 Spectral Analyser (Sony, Tokyo). The mean fluorescence intensity (MFI) of CD42b within a gate region of CD61-positive cells was then measured.

### Platelet PAC-1 binding

PAC-1, which recognizes the activated form of GPIIb/IIIa, was purchased from BD Bioscience. PAC-1-binding platelets were prepared according to the manufacturer’s instructions. In brief, FITC-conjugated PAC-1 and PerCP-conjugated CD61 were mixed and incubated with UV-illuminated platelets for 20 min. Platelets were fixed with cold 1% PFA and stored at 4°C for at least 30 min. Platelets were then evaluated using the SA3800 Spectral Analyser. MFI of FITC gated on CD61-positive cells was determined as the amount of PAC-1 binding.

### Surface expression of CD62P

Surface expression analysis of CD62P (granule membrane protein, GMP-140), which is expressed on the platelet surface when activated platelets release granules, was carried out as previously described [29]. In this study, TRAP-stimulated platelets were used as positive controls for full activation of platelets. Briefly, PCs were diluted with type AB normal plasma at a final platelet concentration of 3.0 × 10^5^/μL, and then activated with TRAP (final concentration, 20 μM) as a platelet activating agonist for 2 min. The activated platelets were then fixed with 1% PFA, washed with PBS, membrane filtered (2-μm pore size), and then labelled with both PerCP-conjugated anti-CD61 and PE-conjugated anti-human CD62P antibodies (or PE-conjugated mouse IgG as a negative control) by incubating for 20 min at room temperature in the dark. The samples were then washed with PBS and evaluated using the SA3800 Spectral Analyser. The ratio of CD62P-positive cells to CD61-positive cells was then determined.

### Agonist-induced platelet aggregation

Prior to the aggregation study, platelets were adjusted to a final platelet concentration of 2.0 × 10^5^/μL by adding type AB normal plasma. Calcium chloride (final concentration, 4 mM) was added to an assay cuvette containing 180 μL of platelet-rich plasma just before the addition of the agonist. Agonist-induced platelet aggregation was then assessed using a Hematoracer 912 (LMS, Tokyo) for 7 min using plasma as a reference for 100% transmittance. The maximum observed % transmittance was defined as platelet aggregation.

### Platelet adherence and aggregation under flow conditions

Collagen tips were prepared by following the manufacturer’s instructions (Cellix Ltd., Dublin, Ireland). In brief, 50 μL of the 50 μg/mL collagen reagent Horm (Moriya Sangyo) was loaded on a Vena8 microfluidic biochip (height, width, and length of each channel were 0.1, 0.4, and 28 mm, respectively) (Cellix Ltd.), incubated at 37°C for at least 1 h, and then washed with PAS-E (formerly PAS-IIIM; the composition of this solution is described in [29]) before use. Platelet samples were diluted to a platelet concentration of 5.0 × 10^4^/μL by adding AB plasma, 3 mM calcium chloride, and 10 μM mepacrine (Wako Chemical, Osaka). This platelet solution was then immediately loaded onto a collagen-coated biochip at a constant flow rate of 3.2 or 32 μL/min using a syringe pump, and surface coverage was determined using an IX71 fluorescence microscope (Olympus, Tokyo) with Metamorph v7.7.7 imaging software (Molecular Devices, Tokyo).

### Measurement of absorbance of PCs at 265 nm

The number of platelets was determined using a cell counter (Sx-800; Sysmex). A 10-μL aliquot of the PC product was diluted with a pre-sterilized PAS-E solution by 100-fold, and the absorbance at 265 nm was measured using a UV-1800 spectrophotometer (10-mm path length) (Shimadzu, Kyoto). PAS-E solution without PC was used as a reference.

### Statistical analysis

The significance of differences between various samples was determined by paired *t* tests with two tails. P values were obtained using Microsoft Excel 2013.

## Results

### Bacterial inactivation by UV-LED irradiation

**Fig 2:**
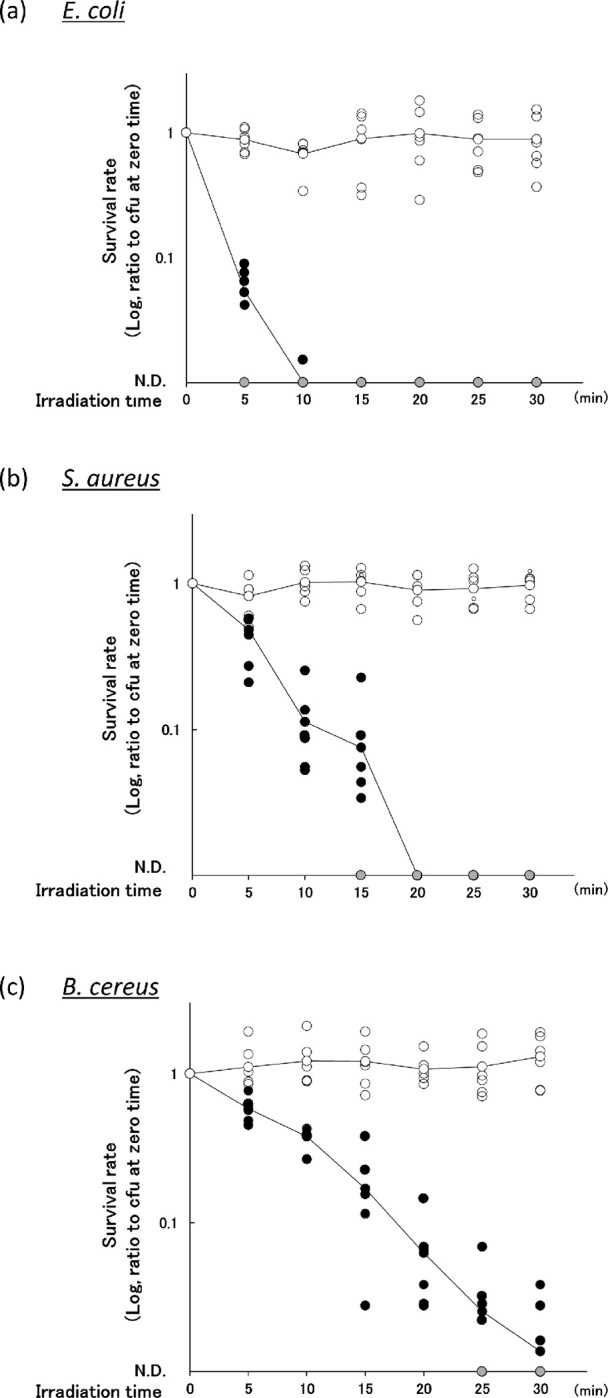
UV-LED inactivation of bacteria in the plasma-PC milieu. PC samples (2.75 mL) inoculated with 6 × 10^3^–2 × 10^4^ cfu of (a) *E*. *coli*, (b) *S*. *aureus*, and (c) *B*. *cereus* were irradiated with UV-LED at 265 nm up to 30 min. Samples (100 μL) were taken every 5 min and incubated overnight for colony count determination. Mean cfu of (a) *E*. *coli*, (b) *S*. *aureus*, and (c) *B*. *cereus* from 100-μL PC samples before irradiation were 50.8, 28.5, and 38.8, respectively. The survival rate was determined as the cfu at each sampling time relative to the cfu before irradiation. The control samples (without UV-LED irradiation) and UV-LED-irradiated samples are displayed as open and closed circles, respectively. The mean value of six independent experiments is shown as a solid line. Those not-detected (ND) are shown as grey circles, and the limits of detection for (a) *E*. *coli*, (b) *S*. *aureus*, and (c) *B*. *cereus* were 0.017, 0.014, and 0.016, respectively. Based on the mean value of 58.6 (cm^-1^) as the 265 nm absorbance for the PC samples tested (n = 520), the average irradiance (adjusted using the Beer-Lambert law) was 0.0156 mW/cm^2^. Thus, on average, irradiation times of 5, 10, 15, 20, 25, and 30 min correspond to fluence values of 4.6, 9.2, 13.9, 18.6, 23.5, and 28.5 mJ/cm^2^, respectively.

All UV-LED irradiations were performed with the bench-scale UV-LED setup shown in Fig 1. Specific details of the setup are described in the Methods section. In control experiments without UV-LED irradiation (open circles; Fig 2A–2C) of the three bacterial species (*E*. *coli*, *S*. *aureus*, and *B*. *cereus*), a 100-μL aliquot taken from the spiked PC mixture at each time interval formed a total of 10–80 colonies on agar plates. Thus, the number of bacteria in the control samples was relatively stable for 30 min after inoculation. With UV-LED irradiation (closed and grey circles; Fig 2), *E*. *coli* was the most rapidly inactivated among the bacterial species; about 1 log (90%) inactivation was achieved after 5 min of irradiation, corresponding to a fluence of 4.6 mJ/cm^2^, and fell below the limit of detection after 10 min (9.2 mJ/cm^2^). Meanwhile, about 10 min of irradiation (9.2 mJ/cm^2^) was needed to produce a 1 log reduction in *S*. *aureus*, and 20 min (18.6 mJ/cm^2^) was needed to reach the limit of detection. In *B*. *cereus*, a gradual inactivation with increasing time of irradiation was observed; however, the detection limit was not reached during the 30-min exposure (28.5 mJ/cm^2^).

### Platelet counts and aggregation after UV-LED irradiation

**Fig 3:**
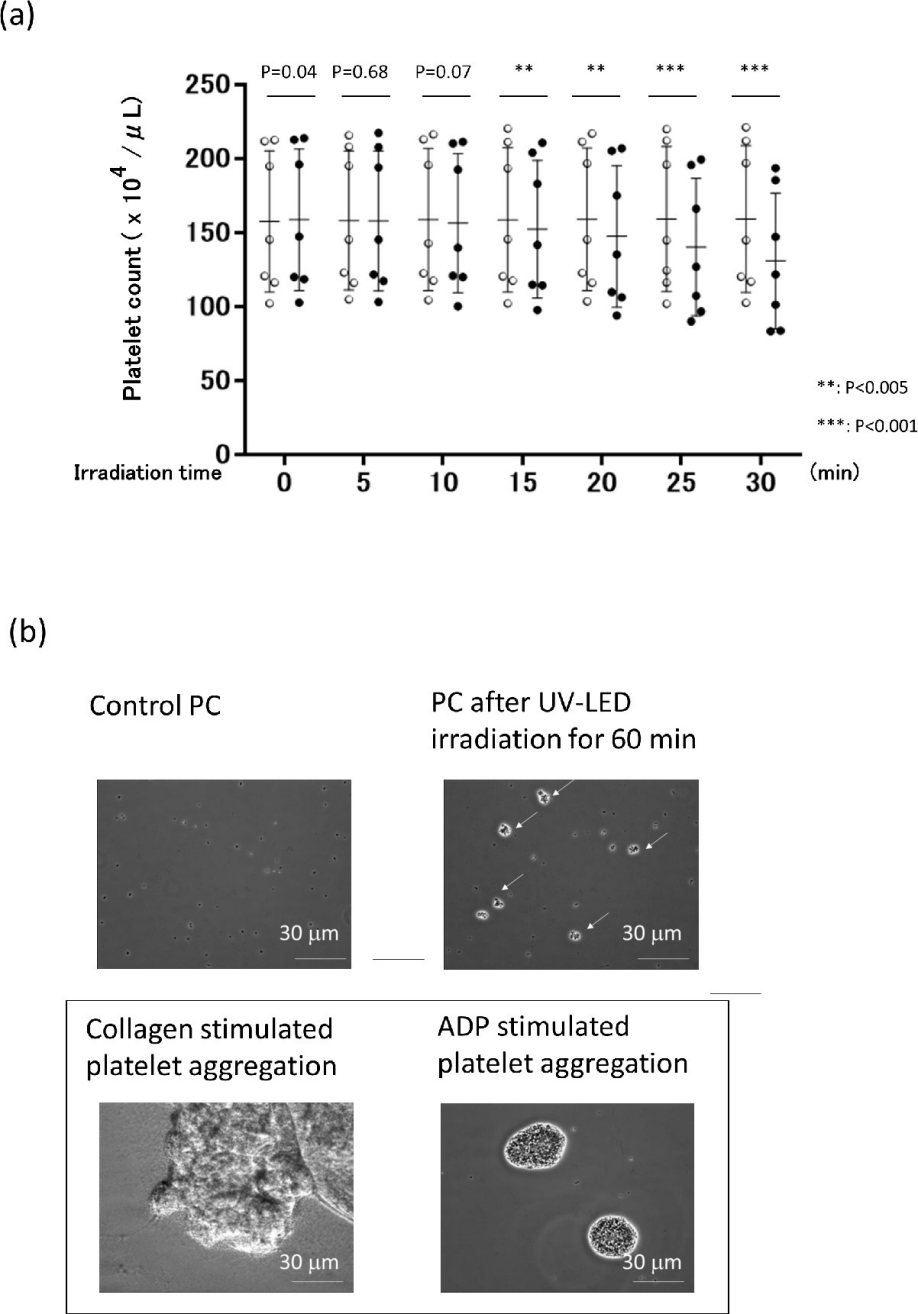
Effects of UV-LED irradiation on platelet counts and aggregation. (a) Seven independent experiments of control (non-irradiated) and UV-LED-irradiated samples are displayed as open and closed circles, respectively. Mean values ± SD are shown as one long and two short horizontal bars. Based on the mean absorbance of PC samples, irradiation times of 5, 10, 15, 20, 25, and 30 min correspond to fluence values of 4.6, 9.2, 13.9, 18.6, 23.5, and 28.5 mJ/cm^2^, respectively. (b) After a 60-min irradiation, PCs were fixed with 1% paraformaldehyde (PFA). For preparation of agonist-induced aggregation, PCs were incubated with 10 μg/mL collagen and 20 μM ADP for 5 min and subsequently fixed with PFA (inset). A representative image from three independent experiments is shown. Arrowheads indicate UV-LED-induced aggregates.

In control samples without irradiation (open circles; Fig 3A), platelet counts were stable for 30 min. In samples with UV-LED irradiation, platelet counts were stable and statistically similar during the first 10 min (P>0.005), followed by a gradual and significant decrease (P<0.005). The differences in platelet counts between samples with and without UV-LED irradiation for 15, 20, 25, and 30 min were 4, 7, 12, and 18%, respectively (Fig 3A). Microscopic observation revealed no platelet aggregates within the first 30 min of exposure to UV-LED. However, irradiation for 60 min showed the presence of fine aggregates (Fig 3B), which were distinctly different in shape and size from those generated by agonist-induced platelet aggregation (described below and shown in Fig 3B inset).

### Changes in platelet surface molecules after UV-LED irradiation

**Fig 4:**
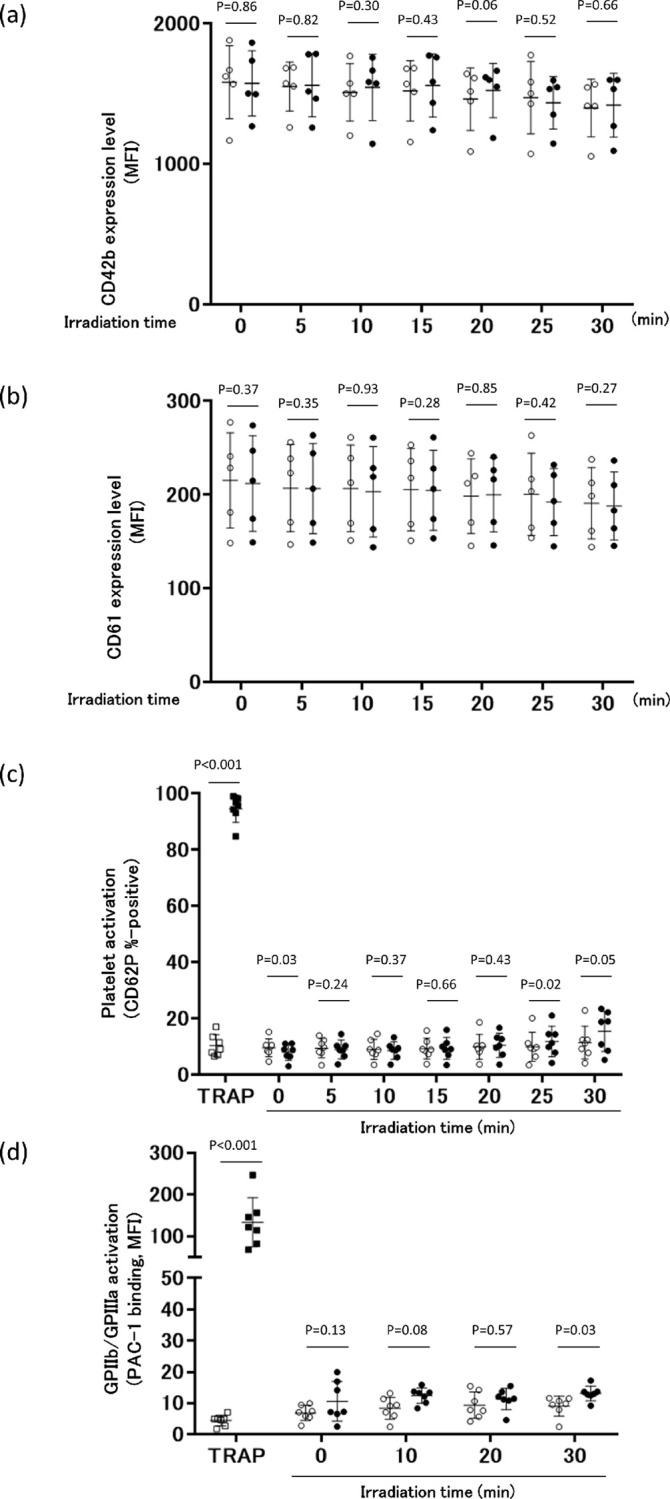
Effect of UV-LED irradiation on platelet surface molecules. Surface expressions of (a) CD42b, (b) CD61, (c) the activated form of GPIIb/IIIa, and (d) CD62P were measured. Five (a, b) and seven (c, d) independent experiments of the control (non-irradiated) and UV-LED-irradiated platelets are displayed as open and closed circles, respectively. Mean values ± SD are shown as one long and two short horizontal bars. (a, b, d) Based on the mean absorbance of PC samples, irradiation times of 0, 5, 10, 15, 20, 25, and 30 min correspond to fluence values of 0, 4.6, 9.2, 13.9, 18.6, 23.5, and 28.5 mJ/cm^2^, respectively. (c) Irradiation times of 0, 10, 20, and 30 min correspond to fluence values of 0, 9.1, 18.3, and 27.7 mJ/cm^2^, respectively.

Fig 4 shows the UV-LED irradiation-induced changes in the surface expression of CD markers on platelets, which are essential for platelet activation and aggregation. The surface expression (shown as MFI) of CD42b was largely unchanged over the 30-min experimental period with or without irradiation (Fig 4A). Similarly, the expression of CD61 (Fig 4B) was relatively stable for 30 min with or without the irradiation. Platelet GPIIb/IIIa activation was measured by PAC-1 binding (Fig 4C), which reflects the amount of activated platelet GPIIb/IIIa. GPIIb/IIIa activation was maintained at resting levels during the 30-min irradiation period. In comparison, TRAP-stimulated normal platelets exhibited a clear increase in the activated form of GPIIb/IIIa. Platelet activation was also measured by determining the proportion of CD62P-positive platelets (Fig 4D). Similarly, TRAP-stimulated normal platelets exhibited a large increase in CD62P staining, which was assumed to represent a fully activated state (100%). Moreover, irradiation did not significantly increase CD62P-positive signals over the 30-min treatment period, and both experimental groups remained within normal values.

### Changes in platelet aggregation after UV-LED irradiation

**Fig 5:**
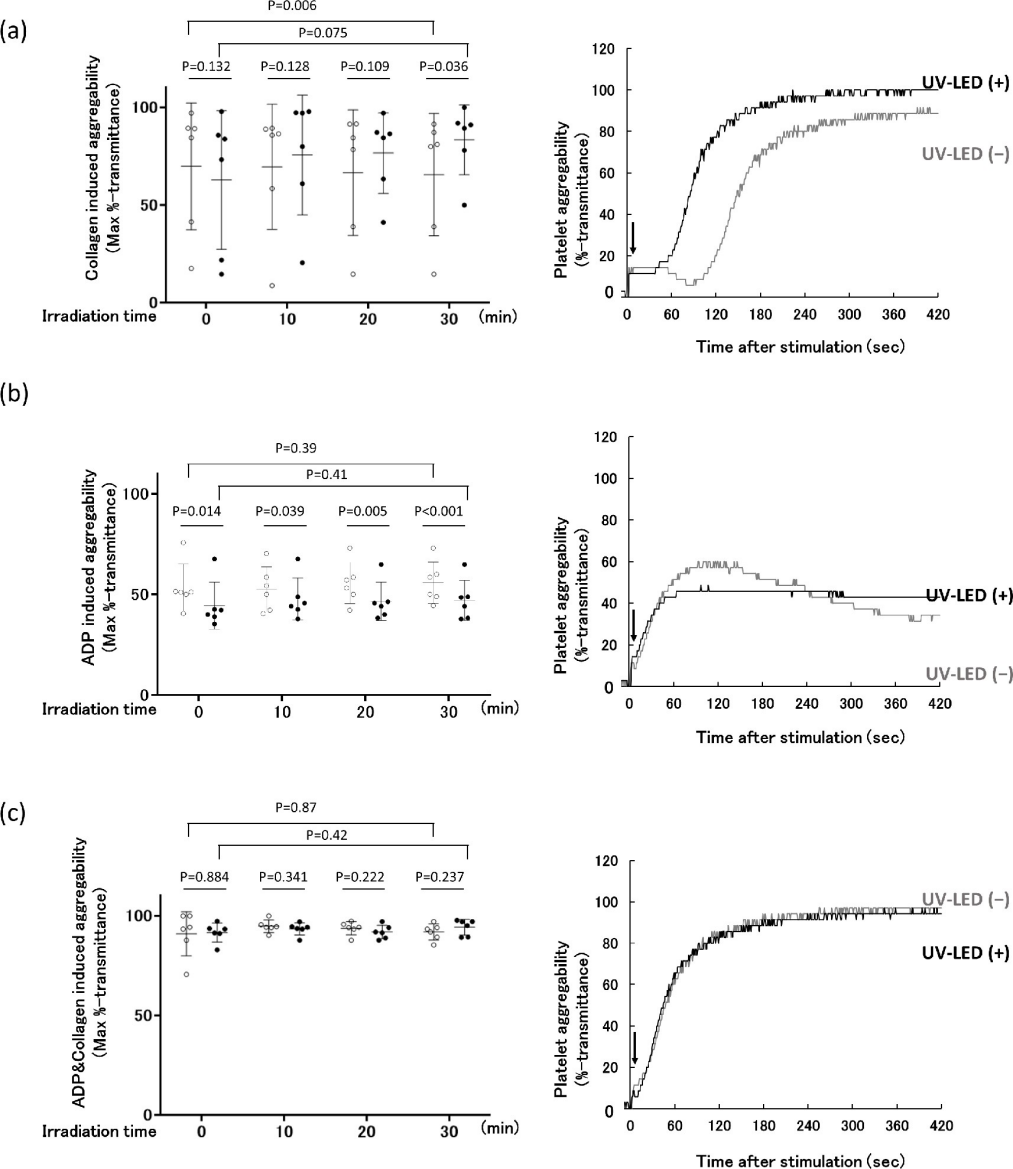
Effect of UV-LED irradiation on agonist-induced platelet aggregation. (a) Platelet aggregation induced by collagen (final concentration, 5 μg/mL), (b) ADP (final concentration, 20 μM), and (c) a mixture of ADP and collagen (final concentrations of 5 μM and 2.5 μg/mL, respectively) are shown. Six independent experiments of control (non-irradiated) and UV-LED-irradiated samples are displayed as open and closed circles, respectively. Mean values ± SD are shown as one long and two short horizontal bars. Based on the mean absorbance of PC samples, irradiation times of 10, 20, and 30 min correspond to fluence values of 9.1, 18.3, and 27.7 mJ/cm^2^, respectively. (a-c right panels) PCs were irradiated with UV-LED for 30 min. Platelets were then diluted as described in the Materials and Methods and stimulated with agonists at time 0 (arrow). Aggregation curves of irradiated (UV-LED (+)) and non-irradiated (UV-LED (−)) platelets are displayed as black and grey lines, respectively. Representative data are shown.

Fig 5 shows the changes in agonist-induced platelet aggregation after UV-LED irradiation. Collagen-induced platelet aggregation (Fig 5A, left) was slightly enhanced with a 30-min irradiation. A representative platelet aggregation experiment is shown in Fig 5A (right), which demonstrates that the lag phase of the platelet response is shortened in the 30-min irradiation sample as compared to the control (no irradiation). ADP-induced platelet aggregation (Fig 5B, left) indicated that the maximum aggregation of irradiated platelets was slightly lower than that of control platelets at the beginning of the experiment, but not significantly so. After 20 min, the maximum platelet aggregation was significantly different between the control and irradiated platelets. Fig 5B (right) shows a representative ADP-induced platelet aggregation experiment using platelets obtained at 30 min with and without irradiation. The control showed modest disaggregation beginning at 120 s after ADP stimulation; however, this was not observed in the irradiated platelets, and the extent of maximum aggregation was slightly less than in the absence of irradiation (control). Platelet aggregation was then examined with both ADP and collagen (Fig 5C, left); no significant differences were observed between the platelets with and without UV-LED irradiation for 30 min. A representative experiment is shown in Fig 5C (right). The ADP-induced aggregation pattern of UV-LED irradiated-platelets was comparable with that of platelets treated with Theraflex UV system [30].

### Changes in platelet adhesion and aggregation to collagen under flow after UV-LED irradiation

**Fig 6:**
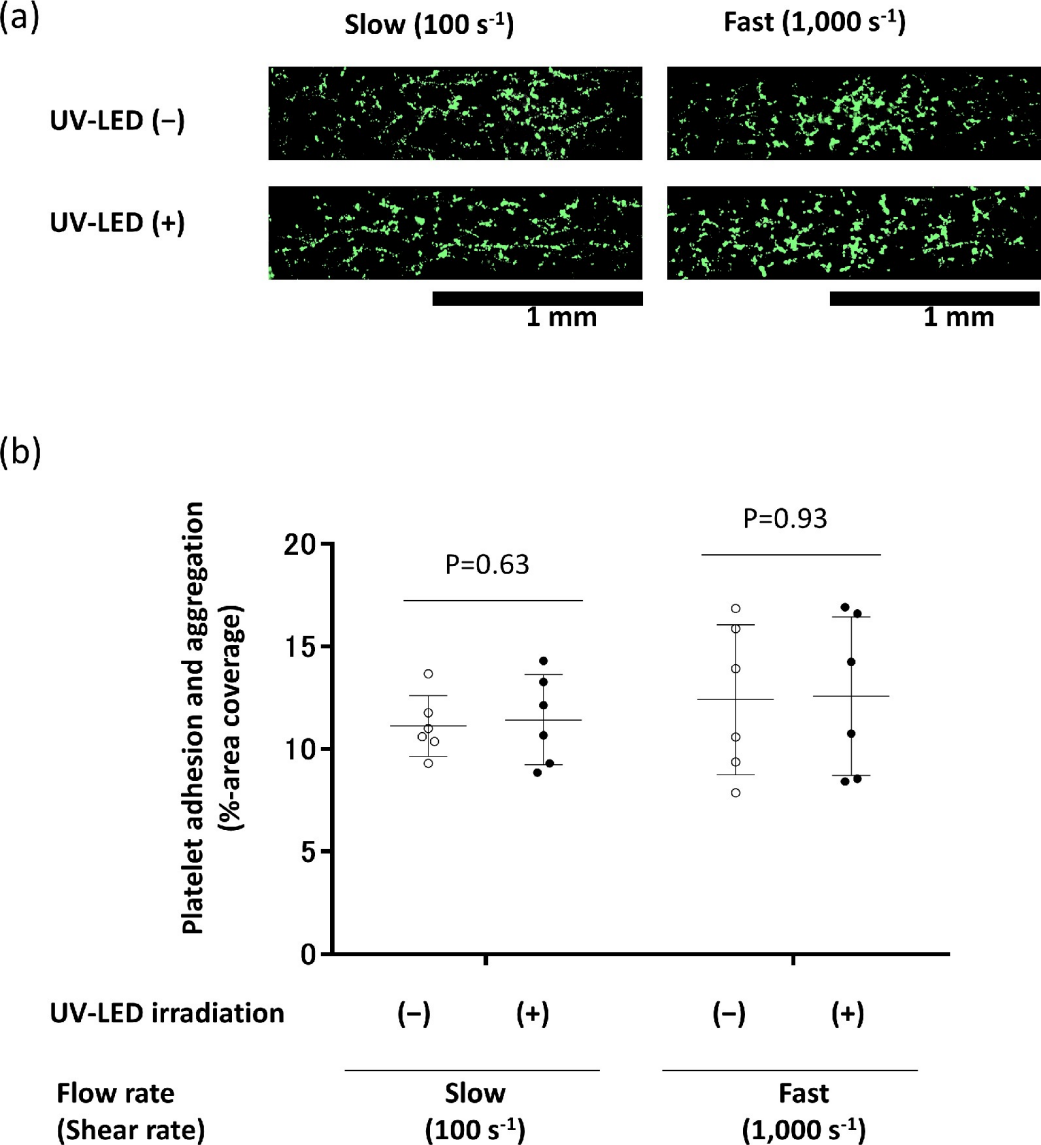
Effect of UV-LED irradiation on adhesion and aggregation to collagen under flow conditions. PCs were irradiated with UV-LED for 30 min. Non-irradiated (UV-LED (−)) and UV-LED-irradiated (UV-LED (+)) platelets were loaded using low (100 s^-1^) and high (1,000 s^-1^) shear rates, and images were obtained after 5 and 2 min of flow, respectively. (a) Representative images from six independent experiments are shown. (b) Six independent experiments of control (non-irradiated, ((−)) and UV-LED-irradiated (+) samples are displayed as open and closed circles, respectively. Mean values ± SD are shown as one long and two short horizontal bars.

We analysed platelet function under two flow conditions, 100 s^-1^ and 1,000 s^-1^, using collagen-coated microchannels. These experiments were each repeated six times using independent platelet samples. The percent coverage of fluorescence, a reflection of both platelet adhesion and aggregation, was measured in an area 8 mm inside of the microchannel inlets (Fig 6A). These results show that platelet adhesion to collagen and aggregation under the two flow conditions were almost indistinguishable between the two treatment groups (Fig 6B). This indicates that platelet adhesion and aggregation were maintained with or without irradiation for 30 min.

### Variation in UV absorbance of PC products

**Fig 7:**
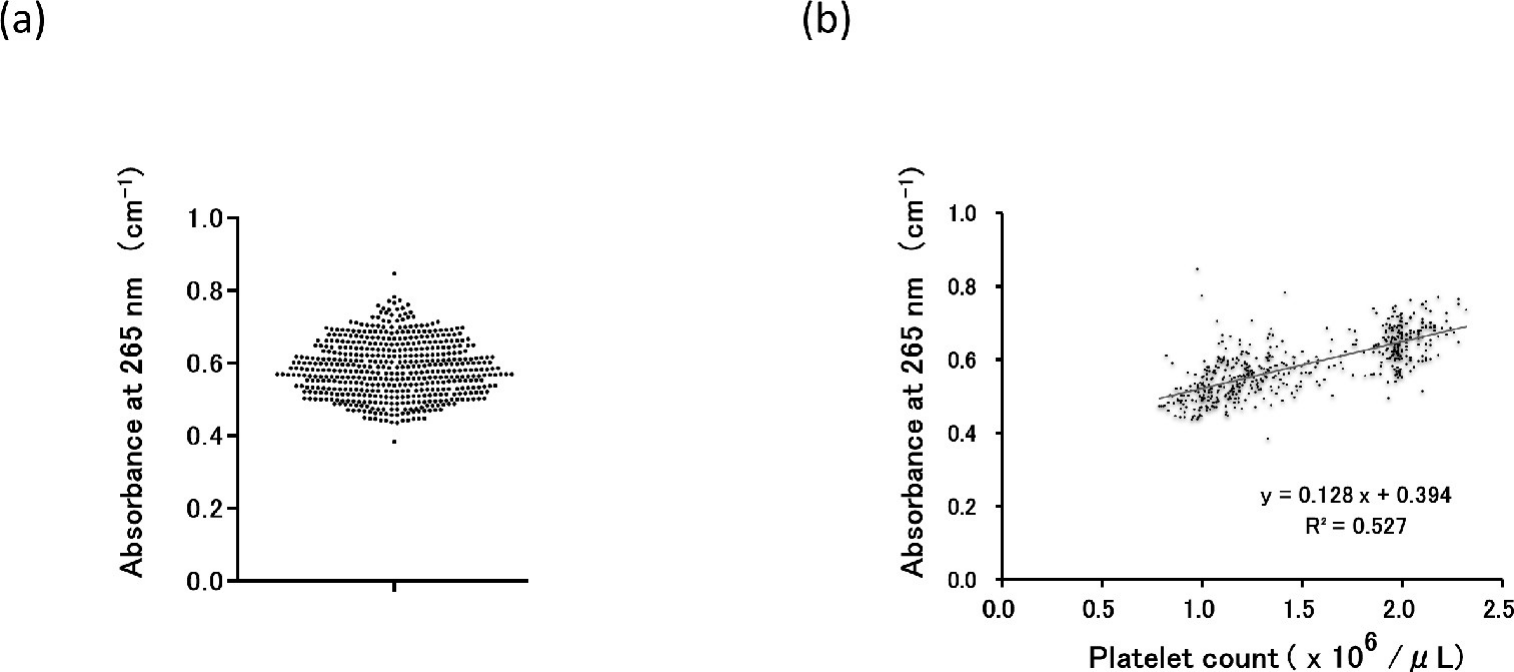
Absorbance (265 nm) of PC samples. PC samples (n = 520; 100-fold dilution) were applied to spectrophotometric analysis to determine absorbance at 265 nm. (a) A dot plot of 520 independent analyses is shown. (b) Linear regression analysis of platelet counts and absorbance at 265 nm.

UV light attenuation due to photon absorption in samples is of significant concern for UV disinfection of PC; thus, the absorbance at 265 nm of PC samples was determined. In total, 520 independent PC products from normal donors were tested after 100-fold dilution with PAS-E solution (Fig 7A). The mean value ± SD of absorbance at 265 nm (at 1:100 dilution) was 0.586±0.077 with a coefficient of variation of 13%. Thus, PC products showed a certain degree of variance in UV absorbance, which is probably due to individual differences among donors. Furthermore, there was a positive linear correlation between absorbance and platelet counts (Fig 7B) (r^2^ = 0.527, P<0.005), indicating that platelets are the major element attenuating UV transmission in PCs.

## Discussion

In the past decade, efforts have been made to develop a single-step disinfection method for PCs by using a conventional low-pressure mercury UV lamp with an emission wavelength of 254 nm, as it works in the absence of photosensitizers [31, 32]. A similar approach with UVC radiation at ≈270 nm has been proposed in bench-scale experiments [33–35] and uses xenon (Xe)-lamp flush through a selective optical bandpass filter for PC disinfection. These methods have some limitations, including safety concerns associated with the use of mercury in low-pressure UV lamps, and the large size and high heat generation associated with Xe-lamps. The use of UV-LEDs would overcome these concerns; however, their application to blood derivatives has not been investigated. This is the first study to evaluate UV-LED irradiation of PCs in a plasma milieu. The results of this study indicated that UV-LED irradiation at 265 nm could reduce bacteria from 50 to 0 cfu/plate after 10 min (9.2 mJ/cm^2^) for *E*. *coli*, and from 30 to 0 cfu/plate after 20 min (18.6 mJ/cm^2^) for *S*. *aureus*. In contrast, the colony count (40 cfu/plate) of *B*. *cereus* decreased in a time-dependent manner but did not reach zero detection even after 30 min of exposure (28.5 mJ/cm^2^), except for in two of six experiments. These findings appear to be in good agreement with previous reports [35].

In Table 2, we summarize our results and those of three other groups who investigated UVC inactivation of PCs using different UV light sources. Briefly, two groups used platelet additive solution (PAS)-PCs with 10 or 35% plasma concentrations, whereas the remaining two including ours used original (non-PAS) PCs. Two used open petri dishes and the other two used closed bags. The intensity of our 265 nm UV-LED (1.05 mW/cm^2^) was in between two others (0.25 and 7~8 mW/cm^2^). The differences come partly from the differences in the distance between the light source and the target objects and depth of the objects. Of note, the relative transparency of PCs under UVC irradiation is an important factor in improving disinfection efficiency and reducing processing time. For this purpose, Terpstra et al. used an orbital shaker, whereas Theraflex® utilizes a bilateral irradiation method in plastic bags together with continuous agitation, leading to a processing time of ≤ 1 min [32, 36]. Meanwhile, Abe et al. used a unique flow path-irradiation plastic bag, sandwiched by two U330/250 nm filters, with a peristaltic pump linked to a collection bag [34], but we utilized a continuous stirring method. Each experiment was performed with different conditions; thus, a direct comparison between our study and other reports is difficult. However, the values of fluence, which reflect experimental conditions including light intensity and the light transparency of objects, are helpful for a qualitative comparison.

**Table 2:** Characteristics of UV irradiation techniques. Data extrapolated from reports.

	#36 (Terpstra et al.)				#32 (Theraflex)	#34 (Abe et al.)	This manuscript			
Technique and equipment										
Light source	low pressure mercury arc				low pressure mercury arc	Xe flash lamp with band-stop filter U330/250	LED			
Emission line	254 nm				254 nm	185 to 320 nm.	265 nm			
Intensity	0.25 mW/cm^2^				7 to 8 mW/cm^2^	1.9	1.05 mW/cm^2^			
						J/cm^2^/1 shot				
Distance from lamp to sample	variable				variable	50 mm	17.5 mm			
Sample mixing	orbital shaker				built-in orbital agitator	rocking shaker	continuous stirring			
Exposure time	0–400 s	50 s	100 s	200 s	20–30 s	780 s	300–1,800 s	600 s	1,200 s	1,800 s
Dose (intensity × exposure time)		125 J/m^2^	250 J/m^2^	500 J/m^2^	2,000 J/m^2^	4900 J/m^2^		6,300 J/m^2^	12,600 J/m^2^	18,900 J/m^2^
Fluence		not determined	not determined	not determined	not determined	not determined		9.2 mJ/cm^2^	18.6 mJ/cm^2^	28.5 mJ/cm^2^
Platelet concentrates										
Residual plasma/PAS	10% plasma/90% PAS				35 ± 5% plasma/65 ± 5% PAS	100% plasma	100% plasma			
Sample volume	5 mL				325–375 mL	191.5–252.1 mL	2.75 mL			
Container	open petri dish				plastic illumination bag	culture bag	open petri dish			
	(84 mm in diameter)						(35 mm in diameter)			
Suspension depth	1 mm				variable	variable	5 mm			
Bacteria reduction rate		>3 log	>4 log	>4 log	>4 log	not determined		>2 log	>2 log	>2 log
		*S*. *aureus*	*S*. *epidermidis*	*S*. *epidermidis*	*B*. *cereus*, *S*. *marcescens*	no bacterial growth was observed except in one of 16 experiments	(LOD: limit of detection)	*E*. *coli* (reach LOD)	*E*. *coli* (reach LOD at 10 min)	*E*. *coli* (reach LOD at 10 min)
		*E*. *coli*	*S*. *aureus*	*S*. *aureus*	*C*. *perfringens*, *S*. *aureus*			Approx. 1 log	*S*. *aureu*s (reach LOD)	*S*. *aureus* (reach LOD at 20 min)
		Approx. 2.2 log	*E*. *coli*	*E*. *coli*	*E*. *coli*, *S*. *epidermidis*	*S*. *aureus* 0.19 cfu/mL		*S*. *aureus*	Approx. 1.2 log	Approx. 1.9 log
		*S*. *epidermidis*	Approx. 2 log	Approx. 3 log	*E*. *cloacae*, *P*. *aeruginosa*	*S*. *dysgalactiae* 0.29 cfu/mL		Approx. 0.4 log	*B*. *cereus*	*B*. *cereus*
		Approx. 1.8 log	*B*. *cereus*	*B*. *cereus*	*K*. *pneumoniae*, *P acnes*			*B*. *cereus*		
		*B*. *cereus*								

As for platelet function, it has been shown that the function can deteriorate to varying degrees after UV irradiation, regardless of the wavelength. In fact, after treatment of PCs with UVA-amotosalen or UVA/B-riboflavin, platelets show slightly less aggregation than untreated samples [37]. In contrast, platelets prepared with UVC at 254 nm irradiation have been reported to maintain their haemostatic function immediately following treatment, and in fact show enhanced platelet aggregation [38]. As an explanation for this phenomenon, 254 nm irradiation of platelets may induce a conformational change in the platelet glycoprotein (GP) IIb/IIIa (integrin αIIbβ3) that leads to its activation, presumably through a photochemical reduction of intramolecular disulphide bonds. Since activated platelet GPIIb/IIIa functions as a receptor for the binding of fibrinogen and von Willebrand factor, both of which are essential for platelet thrombi formation, Feys et al. [20] reported that UVC (254 nm)-treated platelets are primed for activation, increasing the consumption of energy and reaching exhaustion faster. However, in this study we observed that after UV-LED irradiation at 265 nm for 30 min, the surface expression markers of CD42b (GPIIb), CD61 (GPIb), and CD62P were maintained within normal ranges. More importantly, the degree of PAC-1 binding, an indicator of activated platelet GPIIb/IIIa receptor, was almost indistinguishable between platelets with and without UV-LED irradiation. Furthermore, despite the slightly enhanced collagen-induced platelet aggregation, platelet adhesion to a collagen surface under flow conditions was unchanged after UV-LED irradiation for 30 min. These results indicate that platelet activation, if any, is minimized by UV-LED irradiation for 30 min under our experimental conditions. In contrast, a 60-min exposure raised visible platelet aggregation (Fig 3B). Long-term exposure might lead to platelet activation and therefore should be avoided. We summarize the functions of platelets treated with UVC irradiation-based pathogen inactivation techniques in Table 3.

**Table 3:** Effect of UV irradiation techniques on platelets functions. Data extrapolated from reports.

	# 30 (Johnson et al.)	#31 (Mohr et al.)	#34 (Abe et al.)	#35 (Abe et al.) *	This manuscript		
Technique and equipment	Theraflex	Theraflex	Xe flash	Xe flash	265 nm UV LED		
Dose (intensity × exposure time)	2,000 J/m^2^	4,000 J/m^2^	4,900 J/m^2^	9,000 J/m^2^	6,300 J/m^2^	12,600 J/m^2^	18,900 J/m^2^
Platelet concentrates							
Residual plasma/PAS	30% plasma / 70% PAS	35% plasma / 65% PAS	100% plasma	40% plasma / 60% PAS	100% plasma		
Platelet functions							
platelet count	*ns*	*ns*	*ns*	*Significantly decreased*	*ns*	*Slightly decreased*	*Significantly decreased*
CD42b expression	*ns*		*ns*	*Significantly decreased*	*ns*	*ns*	*ns*
CD61 expression	*ns*				*ns*	*ns*	*ns*
CD62P expression	*ns*	*Significantly enhanced*	*ns*	*Significantly enhanced*	*ns*	*ns*	*ns*
PAC-1 binding	*Significantly increased*		*ns*	*Significantly increased*	*ns*	*ns*	*Slightly increased*
Collagen induced aggregability	*ns*	*Partly high*	*ns*	*ns*	*ns*	*ns*	*Slightly high*
Adhesion and aggregation under flow							*ns*

* They collected data at day 1 after treatment.

*ns*: The difference between untreated platelets and treated platelets was not significant. Blank column: Not mentioned.

In 2017, Rebulla et al. reported the results of two noninferiority, randomized, controlled trials that were conducted in parallel comparing the safety and efficacy of platelets treated with two commercial pathogen-reduction technologies, Intercept (113 treated vs. 115 control patients) or Mirasol (99 treated vs. 97 control patients), compared to standard platelets [39]. Mortality was not significantly different between these two patient groups, with treated or untreated platelets. However, the transfusion of platelets treated with either of two pathogen reduction techniques resulted in a lower increment of platelet count as compared to the untreated platelets. Thus, to obtain the same value of Corrected Count Increment (CCI) after PC transfusion as for their untreated counterparts, Intercept and Mirasol treatments required 54% and 34% more platelets, respectively, indicating the need for more platelet infusions once a bactericidal procedure is introduced [39]. Although the two commercially available pathogen reduction technologies have achieved very high inactivating capacity, the lifespan of transfused platelets is shortened, which results in shorter intervals between platelet transfusions and an increased number of platelet transfusions per patient [40]. Likewise, the Theraflex UV-platelet system also achieves high inactivating capacity, and the recovery and survival rates of treated platelets are comparable to the aforementioned two pathogen-reduced platelets [41]. According to a US report in 2016 [42], a loss of 20% of plasma potency and 30% of platelet potency due to pathogen reduction is assumed to be related to 400 extra trauma deaths each year. This indicates that there might be situations in which pathogen reduction technology may lead to more deaths than it prevents due to the reduction of recovery. In this regard, LED-UVC 265 nm inactivation technology might have the following advantages: minimal damage to platelet function; less labour than the current complex pathogen reduction methods including bag changes, volume loss, and dilution effects; and no requirement for photosensitizers when processing PC preparations. Presently, however, we are unable to address all of these issues because of our current experimental design, but they will be addressed in future studies.

We believe that the balance between the efficacy of pathogen reduction and the extent of platelet activation/damage is important. In this regard, it is notable that the FDA has recently issued guidance for bacterial risk control strategies for blood collection establishments and transfusion services to enhance the safety and availability of platelets for transfusion [15]. The recommended strategies are either single-step or two-step; the latter ones are composed of primary and secondary bacterial detection testing, i.e., culture and rapid bacterial detection tests. In two-step strategies, the second step remedies the weak point of the primary step, i.e., the incomplete ability of bacterial detection. Neither bacterial culture nor rapid bacterial detection tests affect platelet function. Consequently, some two-step strategies allow for a 7-day expiration date. In this study, we demonstrated that in certain conditions, UV-LED achieves significant though not overwhelming bacterial reduction but does not evoke significant platelet damage. This raises the possibility that combining another strategy, such as bacterial culture, with our UV-LED could provide a novel two-step strategy: a combination of UVC irradiation without a photosensitizer and bacterial culture (e.g. BacT/Alert). We believe this is a promising candidate strategy to control the risk of bacterial contamination of platelet products and simultaneously achieve 7-day storage because of the low damage to platelets. Disinfection methods for PCs should be evaluated for their inactivation efficiency as well as their effect on the stability of PCs during storage. In this regard, the limitations of our study are that the experiments were performed at bench-scale, and treated samples were not packed for storage and stability evaluation. It should be noted that sensitivity to UV irradiation differs among species, and only three bacterial species at low doses were tested in this study. Additionally, as for delayed bacterial growth, the degree of attenuated bacterial recovery after UV-LED irradiation, by photoreactivation and dark repair, needs to be examined in a future study.

Currently, disinfection by UV LEDs is limited by the relatively low radiant flux compared to mercury LP lamps. However, significant progress has been made in the development of photonic devices and the use of nanostructured materials for LEDs due to their unique geometry [43, 44]. Nanostructures in small dimensions can be integrated into a variety of technological platforms, offering novel physical and chemical properties for high-performance light-emitting devices. By alloying GaN with AlN materials, the emission of AlGaN-based LEDs can be tuned to cover almost the entire UV spectral range (210–400 nm) [45], and this has allowed insight into the photobiological effects of these wavelength bands. In a recent study, the inactivation kinetics of *E*. *coli* were determined at four different central LED wavelengths (265, 275, 285, and 295 nm), and for 265 nm, higher DNA damage was observed, whereas for 285 and 295 nm, higher oxidative stress was observed [46]. In this regard, 265 nm might be suitable for PC irradiation, because PC contains many proteins and anucleate cells, i.e. platelets.

We also determined the variation of absorbance at 265 nm for 520 PCs obtained from different normal donors, following 100-fold dilution with saline/PAS, which showed a value (mean ± SD) of 0.586±0.077, and a positive linear relationship between the absorbance and platelet counts. These results show that the lower the plasma concentration in PCs, the higher transmittance at 265 nm, unambiguously indicating that UV treatment would work better with diluted-plasma PCs such as PAS-PC or washed PC. Furthermore, variations in absorbance and the coefficient of variation of absorbance at 265 nm are important factors to consider in the development of an irradiation device.

UV-LEDs are very compact devices, and the dimensions of 265 nm UV-LED package used in this study were only 3.5 mm per side. In general, the properties of UV-LEDs (mercury-free, no heat dissipation, long lifetime, no warm-up time, and small size) are well suited to the development of devices for PC disinfection. Further, UV-LED-based irradiation devices can be designed in various configurations, and therefore might be readily incorporated into existing blood processing and/or blood collection machines. Designing such systems will be explored in future work. We believe that UV-LED is a promising as a light source to improve the bacteriological safety of platelet transfusions.

## Supporting information

S1 Fig Colony formation of bacteria. One colony formed by each of three species of bacteria (*E*. *coli*, *S*. *aureus*, and *B*. *cereus*), each containing 10^8^–10^10^ bacteria, was picked and then added to 1 mL of saline. Then the mixtures were diluted 100–10,000-fold with PCs. a) Colony formation in agar plates using PCs spiked with three species of bacteria. Approximately 10^4^–10^6^ cfu of *E*. *coli*, *S*. *aureus*, and *B*. *cereus* were spiked into 10 mL of PCs. Five minutes later, aliquots (100 μL) of PCs were each plated on agar followed by incubation at 37°C or 30°C depending on the bacterial species. Note that no colonies of *E*. *coli* alone were formed under this condition. b) Colony formation of *E*. *coli* spiked into PCs supplemented with EDTA. Approximately 10^4^–10^5^ cfu of *E*. *coli* were spiked into 10 mL of PCs supplemented with EDTA at a final concentration of 5 mmol/L. Thirty minutes later, an aliquot (100 μL) of PCs was plated on agar without EDTA followed by incubation at 37°C overnight. Note that *E*. *coli* formed a number of colonies under this condition.
(PPTX)

S2 Fig The minimal data set. The values used to build Figs 2–7 were shown. The numbers in the left hand are related to the figure number.
(PDF)
