# Supplementary material for: UV light-emitting diode (UV-LED) at 265 nm as a potential light source for disinfecting human platelet concentrates
Source: PLoS One. 2021 May 20;16(5):e0251650. doi: 10.1371/journal.pone.0251650 (PMC8136854; doi:10.1371/journal.pone.0251650)
Supplement: S1 Fig — One colony formed by each of three species of bacteria (E. coli, S. aureus, and B. cereus), each containing 108–1010 bacteria, was picked and then added to 1 mL of saline. Then the mixtures were diluted 100–10,000-fold with PCs. a) Colony formation in agar plates using PCs spiked with three species of bacteria. Approximately 104–106 cfu of E. coli, S. aureus, and B. cereus were spiked into 10 mL of PCs. Five minutes later, aliquots (100 μL) of PCs were each plated on agar followed by incubation at 37°C or 30°C depending on the bacterial species. Note that no colonies of E. coli alone were formed under this condition. b) Colony formation of E. coli spiked into PCs supplemented with EDTA. Approximately 104–105 cfu of E. coli were spiked into 10 mL of PCs supplemented with EDTA at a final concentration of 5 mmol/L. Thirty minutes later, an aliquot (100 μL) of PCs was plated on agar without EDTA followed by incubation at 37°C overnight. Note that E. coli formed a number of colonies under this condition. (PPTX) [file pone.0251650.s001.pptx]

## Slide 1
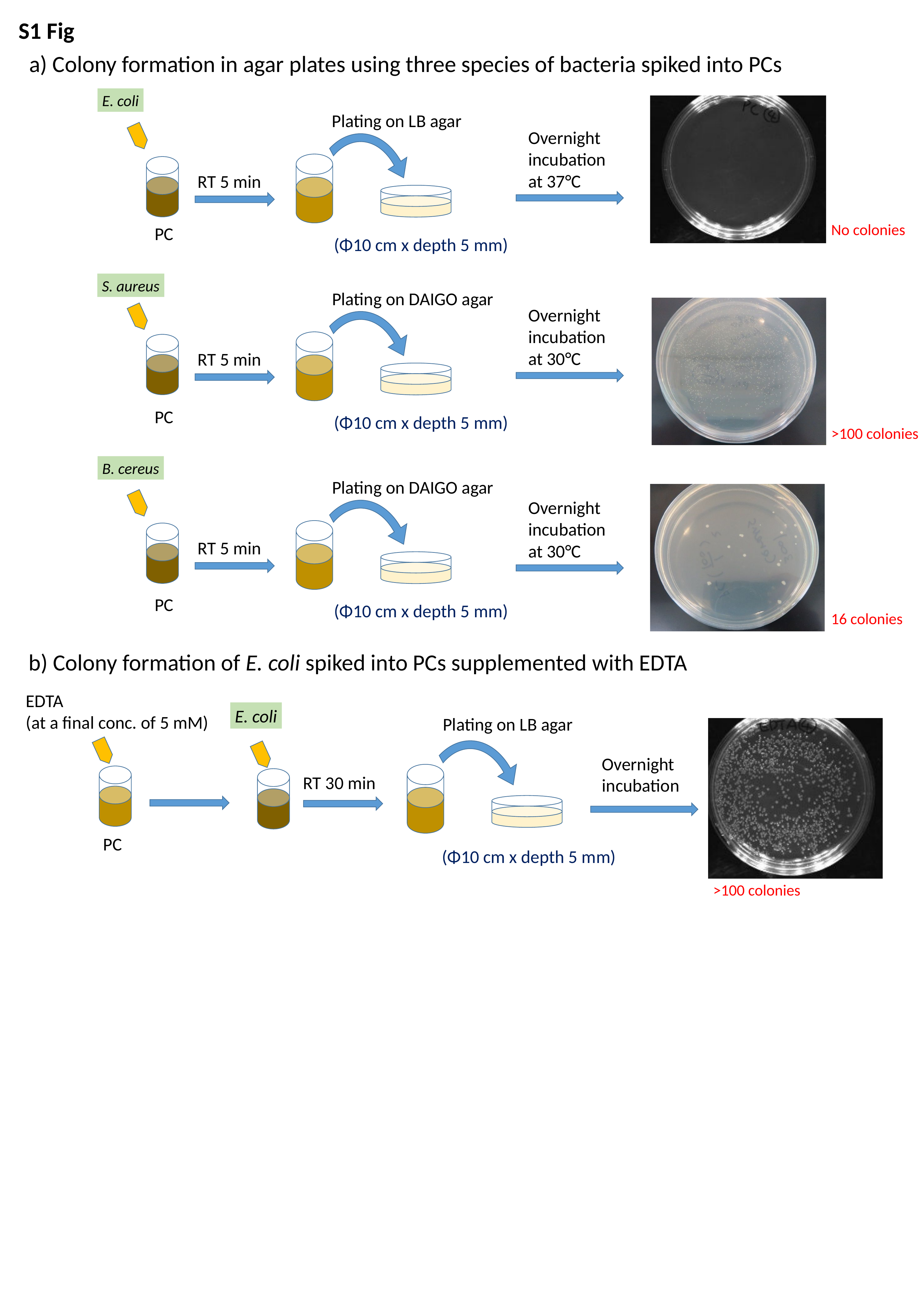

S1 Fig
a) Colony formation in agar plates using three species of bacteria spiked into PCs
E. coli
Plating on LB agar
Overnight
incubation
at 37°C
RT 5 min
No colonies
PC
 (Φ10 cm x depth 5 mm)
S. aureus
Plating on DAIGO agar
Overnight
incubation
at 30°C
RT 5 min
PC
 (Φ10 cm x depth 5 mm)
>100 colonies
B. cereus
Plating on DAIGO agar
Overnight
incubation
at 30°C
RT 5 min
PC
 (Φ10 cm x depth 5 mm)
16 colonies
b) Colony formation of E. coli spiked into PCs supplemented with EDTA
EDTA
(at a final conc. of 5 mM)
E. coli
Plating on LB agar
Overnight
incubation
RT 30 min
PC
 (Φ10 cm x depth 5 mm)
>100 colonies
