# Supplementary material for: UV light-emitting diode (UV-LED) at 265 nm as a potential light source for disinfecting human platelet concentrates
Source: PLoS One. 2021 May 20;16(5):e0251650. doi: 10.1371/journal.pone.0251650 (PMC8136854; doi:10.1371/journal.pone.0251650)
Supplement: S2 Fig — The values used to build Figs 2–7 were shown. The numbers in the left hand are related to the figure number. (PDF) [file pone.0251650.s002.pdf]

S2 Fig. The minimal data set.

Fig. 2

E. coli

Colony count	0 Time	5min	10min	15min	20min	25min	30min
#1 w/o UV	65	60	53	69	60	46	54
#2 w/o UV	26	28	21	37	47	34	40
#3 w/o UV	43	30	31	38	37	39	28
#4 w/o UV	77	52	53	28	46	37	44
#5 w/o UV	43	35	30	58	63	60	58
#6 w/o UV	38	42	13	12	11	19	14
Mean	49	41	34	40	44	39	40
SD	19	13	16	21	19	14	16

Colony count	0 Time	5min	10min	15min	20min	25min	30min
#1 UV irradiated	66	5	1	0	0	0	0
#2 UV irradiated	57	3	0	0	0	0	0
#3 UV irradiated	31	2	0	0	0	0	0
#4 UV irradiated	72	3	0	0	0	0	0
#5 UV irradiated	45	4	0	0	0	0	0
#6 UV irradiated	34	0	0	0	0	0	0
Mean	51	3	0	0	0	0	0
SD	17	2	0	0	0	0	0

Ratio to 0 time	0 Time	5min	10min	15min	20min	25min	30min
#1 w/o UV	100%	92%	82%	106%	92%	71%	83%
#2 w/o UV	100%	108%	81%	142%	181%	131%	154%
#3 w/o UV	100%	70%	72%	88%	86%	91%	65%
#4 w/o UV	100%	68%	69%	36%	60%	48%	57%
#5 w/o UV	100%	81%	70%	135%	147%	140%	135%
#6 w/o UV	100%	111%	34%	32%	29%	50%	37%
Mean	100%	88%	68%	90%	99%	88%	88%

Ratio to 0 time	0 Time	5min	10min	15min	20min	25min	30min
#1 UV irradiated	100%	8%	2%	0%	0%	0%	0%
#2 UV irradiated	100%	5%	0%	0%	0%	0%	0%
#3 UV irradiated	100%	6%	0%	0%	0%	0%	0%
#4 UV irradiated	100%	4%	0%	0%	0%	0%	0%
#5 UV irradiated	100%	9%	0%	0%	0%	0%	0%
#6 UV irradiated	100%	0%	0%	0%	0%	0%	0%
Mean	100%	5%	0%	0%	0%	0%	0

S. aureus

Colony count	0 Time	5min	10min	15min	20min	25min	30min
#1 w/o UV	14	12	14	16	12	11	17
#2 w/o UV	25	15	22	22	14	17	27
#3 w/o UV	57	65	70	65	65	72	61
#4 w/o UV	22	20	29	23	25	24	23
#5 w/o UV	12	6	9	8	9	8	8
#6 w/o UV	22	20	21	28	21	23	17
Mean	25	23	28	27	24	26	26
SD	16	21	22	20	21	23	19

Colony count	0 Time	5min	10min	15min	20min	25min	30min
#1 UV irradiated	18	8	1	1	0	0	0
#2 UV irradiated	22	18	3	5	0	0	0
#3 UV irradiated	59	34	15	2	0	0	0
#4 UV irradiated	38	8	2	0	0	0	0
#5 UV irradiated	11	3	1	1	0	0	0
#6 UV irradiated	23	13	2	1	0	0	0
Mean	29	14	4	2	0	0	0
SD	17	11	5	2	0	0	0

Ratio to 0 time	0 Time	5min	10min	15min	20min	25min	30min
#1 w/o UV	100%	86%	100%	114%	86%	79%	121%
#2 w/o UV	100%	60%	88%	88%	56%	68%	108%
#3 w/o UV	100%	114%	123%	114%	114%	126%	107%
#4 w/o UV	100%	91%	132%	105%	114%	109%	105%
#5 w/o UV	100%	50%	75%	67%	75%	67%	67%
#6 w/o UV	100%	91%	95%	127%	95%	105%	77%
Mean	100%	82%	102%	102%	90%	92%	97%

Ratio to 0 time	0 Time	5min	10min	15min	20min	25min	30min
#1 UV irradiated	100%	44%	6%	6%	0%	0%	0%
#2 UV irradiated	100%	82%	14%	23%	0%	0%	0%
#3 UV irradiated	100%	58%	25%	3%	0%	0%	0%
#4 UV irradiated	100%	21%	5%	0%	0%	0%	0%
#5 UV irradiated	100%	27%	9%	9%	0%	0%	0%
#6 UV irradiated	100%	57%	9%	4%	0%	0%	0%
Mean	100%	48%	11%	8%	0%	0%	0%

B. cereus

Colony count	0 Time	5min	10min	15min	20min	25min	30min
#1 w/o UV	35	36	39	30	35	34	42
#2 w/o UV	20	27	28	24	20	37	38
#3 w/o UV	23	44	48	44	35	35	41
#4 w/o UV	27	23	24	39	23	19	21
#5 w/o UV	60	38	54	43	56	45	46
#6 w/o UV	52	46	47	59	59	47	74
Mean	36	36	40	40	38	36	44
SD	16	9	12	12	16	10	17

Colony count	0 Time	5min	10min	15min	20min	25min	30min
#1 UV irradiated	35	20	15	4	1	1	0
#2 UV irradiated	26	20	11	3	1	0	1
#3 UV irradiated	36	22	14	1	1	0	1
#4 UV irradiated	29	14	11	11	2	2	0
#5 UV irradiated	62	28	24	14	9	2	1
#6 UV irradiated	45	28	12	7	3	1	0
Mean	39	22	15	7	3	1	1
SD	13	5	5	5	3	1	1

Ratio to 0 time	0 Time	5min	10min	15min	20min	25min	30min
#1 UV irradiated	100%	103%	111%	86%	100%	97%	120%
#2 UV irradiated	100%	135%	140%	120%	100%	185%	190%
#3 UV irradiated	100%	191%	209%	191%	152%	152%	178%
#4 UV irradiated	100%	85%	89%	144%	85%	70%	78%
#5 UV irradiated	100%	63%	90%	72%	93%	75%	77%
#6 UV irradiated	100%	88%	90%	113%	113%	90%	142%
Mean	100%	111%	122%	121%	107%	112%	131%

Ratio to 0 time	0 Time	5min	10min	15min	20min	25min	30min
#1 UV irradiated	100%	57%	43%	11%	3%	3%	0%
#2 UV irradiated	100%	77%	42%	12%	4%	0%	4%
#3 UV irradiated	100%	61%	39%	3%	3%	0%	3%
#4 UV irradiated	100%	48%	38%	38%	7%	7%	0%
#5 UV irradiated	100%	45%	39%	23%	15%	3%	2%
#6 UV irradiated	100%	62%	27%	16%	7%	2%	0%
Mean	100%	58%	38%	17%	6%	3%	1%

Fig. 3

PLT count	0 Time	5min	10min	15min	20min	25min	30min
#1 w/o UV	101.9	104.6	104.3	101.9	103.5	101.7	102.3
#2 w/o UV	212.4	207.9	212.9	211.0	211.2	211.9	211.7
#3 w/o UV	194.7	194.9	195.5	193.2	196.5	195.8	196.8
#4 w/o UV	116.1	115.9	117.3	117.3	115.9	116.1	116.7
#5 w/o UV	211.5	215.6	216.1	220.1	216.7	219.7	220.9
#6 w/o UV	120.5	122.9	122.3	120.3	122.7	124.2	120.2
#7 w/o UV	145.0	145.0	142.5	145.4	145.0	144.7	144.6
Mean	157.4	158.1	158.7	158.5	158.8	159.2	159.0
SD	47.7	46.9	48.0	48.8	48.2	48.9	49.6

PLT count	0 Time	5min	10min	15min	20min	25min	30min
#1 UV irradiated	102.5	103.0	100.0	97.7	93.9	89.8	83.7
#2 UV irradiated	212.6	207.5	211.2	203.8	205.0	195.6	193.5
#3 UV irradiated	195.9	193.9	192.3	182.8	174.8	166.0	147.0
#4 UV irradiated	118.4	117.1	120.7	114.2	109.7	107.1	101.1
#5 UV irradiated	213.7	217.1	210.2	210.6	206.7	199.2	185.4
#6 UV irradiated	119.9	121.6	119.8	114.7	106.1	96.5	83.3
#7 UV irradiated	147.2	145.2	139.6	141.6	135.2	126.7	121.5
Mean	158.6	157.9	156.3	152.2	147.3	140.1	130.8
SD	47.8	47.3	47.0	46.4	47.8	46.4	45.9

Fig. 4

CD42b	0 Time	5min	10min	15min	20min	25min	30min
#1 w/o UV	1169.44	1262.41	1203.51	1159.58	1090.55	1074.79	1056.8
#2 w/o UV	1881.88	1684.56	1771.26	1684.28	1614.75	1777.97	1569.45
#3 w/o UV	1625.31	1575.03	1502.84	1572.44	1519.25	1430.01	1415.27
#4 w/o UV	1671.1	1688.96	1576.01	1680.15	1644.43	1586.23	1547.57
#5 w/o UV	1570.35	1556.34	1503.34	1515.12	1448.47	1504	1414.02
Mean	1583.616	1553.46	1511.392	1522.314	1463.49	1474.6	1400.622
SD	259.8418	173.7319	204.1055	215.224	222.5072	258.4547	205.3681

CD42b	0 Time	5min	10min	15min	20min	25min	30min
#1 UV irradiated	1270.81	1260.21	1146.46	1242	1187.3	1147.79	1096.12
#2 UV irradiated	1866.31	1782.77	1758.75	1760.42	1601.23	1549.27	1600.38
#3 UV irradiated	1499.83	1467.11	1576.1	1437.59	1618.56	1353.34	1272.31
#4 UV irradiated	1738.08	1786.03	1667.85	1773.78	1665.65	1598.21	1601.25
#5 UV irradiated	1503.79	1519.01	1585.85	1586.99	1552.64	1536.29	1535.6
Mean	1575.764	1563.026	1547.002	1560.156	1525.076	1436.98	1421.132
SD	231.6822	224.0831	235.707	225.1148	193.1011	186.476	226.6363

CD61	0 Time	5min	10min	15min	20min	25min	30min
#1 w/o UV	241.08	238.63	239.18	236.16	220.14	216.87	212.8
#2 w/o UV	277.37	256.06	261.35	253.49	244.36	263.42	237.9
#3 w/o UV	228.88	223.46	213.24	218.09	212.45	203.91	198.91
#5 w/o UV	148.69	147.06	151.27	151.09	145.54	154.05	144.28
#6 w/o UV	181.27	170.75	169.9	168.84	170.74	165.19	161.61
Mean	215.458	207.192	206.988	205.534	198.646	200.688	191.1
SD	50.73588	46.32751	46.1266	43.89186	39.81615	43.71142	38.02597

CD61	0 Time	5min	10min	15min	20min	25min	30min
#1 UV irradiated	247	244.25	228.5	228.06	226.29	221.05	210.42
#2 UV irradiated	274.21	263.55	261.07	261.48	240.57	232.09	236.6
#3 UV irradiated	215.09	206.59	219.37	206.44	216.69	193.29	183.37
#5 UV irradiated	149.3	149.21	144.02	153.65	146.24	145.2	145.57
#6 UV irradiated	174.74	170.22	163.67	174.27	171.16	170.03	164.41
Mean	212.068	206.764	203.326	204.78	200.19	192.332	188.074
SD	51.0495	48.16069	48.25793	42.73389	39.80373	35.79506	36.20366

CD62P	0 Time	5min	10min	15min	20min	25min	30min
#1 w/o UV	9.99%	9.84%	9.12%	8.64%	9.44%	9.33%	12.50%

CD62P	0 Time	5min	10min	15min	20min	25min	30min
#1 UV irradiated	9.16%	8.91%	9.14%	9.24%	10.46%	11.37%	18.85%

	%-positive	
	No stimul:	TRAP-induced activation
#1	11.65	98.25

#2 w/o UV	8.51%	7.89%	7.46%	7.42%	8.18%	6.52%	7.90%
#3 w/o UV	15.27%	12.37%	12.48%	11.90%	10.52%	11.27%	12.51%
#4 w/o UV	4.83%	3.44%	3.61%	3.87%	3.76%	3.54%	4.19%
#5 w/o UV	10.60%	14.01%	14.62%	15.82%	18.68%	20.22%	22.95%
#6 w/o UV	10.65%	11.13%	8.86%	9.19%	10.38%	9.85%	11.26%
#7 w/o UV	8.15%	8.24%	7.84%	9.00%	9.41%	9.66%	9.32%
Mean	9.71%	9.56%	9.14%	9.41%	10.05%	10.06%	11.52%
SD	3.2%	3.5%	3.6%	3.7%	4.4%	5.2%	5.8%

#2 UV irradiated	6.63%	6.67%	6.26%	7.16%	7.31%	7.95%	8.59%
#3 UV irradiated	11.13%	10.37%	10.33%	11.37%	12.81%	14.55%	23.52%
#4 UV irradiated	3.13%	3.79%	3.70%	3.53%	3.70%	4.32%	5.36%
#5 UV irradiated	11.17%	14.58%	13.41%	16.08%	16.82%	21.15%	22.28%
#6 UV irradiated	8.96%	10.41%	9.54%	10.16%	13.29%	14.45%	19.02%
#7 UV irradiated	6.90%	8.77%	8.74%	8.95%	9.74%	9.95%	10.89%
Mean	8.15%	9.07%	8.73%	9.50%	10.59%	11.96%	15.50%
SD	2.9%	3.4%	3.1%	3.8%	4.3%	5.4%	7.1%

#2	6.69	94.36
#3	7.12	95.77
#4	9.23	84.75
#5	13.62	96.98
#6	17.13	93.13
#7	8	99
Mean	10.49	94.61
SD	3.85	4.81

PAC-1

activated IIb/IIIa	0 min	10 min	20 min	30 min
#1 w/o UV	9.45	6.06	5.65	10.68
#2 w/o UV	7.47	9.4	10.64	9.88
#3 w/o UV	2.81	2.42	4.18	2.41
#4 w/o UV	5.51	7.06	7.83	7.89
#5 w/o UV	9.91	13.14	14.25	9.46
#6 w/o UV	5.92	9.11	7.64	11.52
#7 w/o UV	7.13	11.4	15.44	11.61
Mean	6.26	8.63	9.87	8.58
SD	2.58	4.16	4.79	3.78

PAC-1

activated IIb/IIIa	0 min	10 min	20 min	30 min
#1 UV irradiated	6.58	12.21	11.19	12.79
#2 UV irradiated	7.17	15.86	11.48	12.48
#3 UV irradiated	2.52	8.39	4.63	9.22
#4 UV irradiated	7.24	10.2	10.84	13.66
#5 UV irradiated	14.13	13.27	15.49	17.21
#6 UV irradiated	19.92	13.72	13.65	13.02
#7 UV irradiated	16.9	13.41	12.09	13.68
Mean	12.14	11.80	11.34	13.36
SD	7.14	2.38	4.14	2.84

	No stimul	TRAP-induced activation
#1	4.97	81.83
#2	7.11	67.81
#3	1.78	121.53
#4	2.76	156.6
#5	5.02	114.67
#6	4.62	145.81
#7	4.97	246.99
Mean	4.46	133.61
SD	1.73	59.23

Fig. 5

Collagen 5μg/mL

	0 Time	10min	20min	30min
#1 w/o UV	89	89	91	87
#2 w/o UV	84	89	84	80
#3 w/o UV	89	86	78	81
#4 w/o UV	18	9	15	15
#5 w/o UV	41	59	39	39
#6 w/o UV	97	86	91	91
Mean	70	70	67	66
SD	32	32	32	31

	0 Time	10min	20min		30min
#1 UV irra	98	98		87	89
#2 UV irra	73	80		84	91
#3 UV irra	84	97		86	92
#4 UV irra	15	21		41	50
#5 UV irra	22	61		63	78
#6 UV irra	86	97		97	100
Mean	63	76		77	83
SD	35	31		21	18

ADP 20μM

	0 Time	10min	20min	30min
#1 w/o UV	40	40	53	49
#2 w/o UV	51	42	42	44
#3 w/o UV	76	70	73	73
#4 w/o UV	50	50	50	50
#5 w/o UV	51	59	59	59
#6 w/o UV	51	54	57	60
Mean	53	53	56	56
SD	12	11	10	10

	0 Time	10min	20min		30min
#1 UV irra	43	43		38	38
#2 UV irra	42	38		40	38
#3 UV irra	68	68		65	65
#4 UV irra	35	44		44	44
#5 UV irra	39	49		46	49
#6 UV irra	40	46		46	49
Mean	44	48		47	47
SD	12	10		10	10

ADP+collagen (5μM+2.5μg/mL)

	0 Time	10min	20min	30min
#1 w/o UV	100	100	96	89
#2 w/o UV	93	96	93	93
#3 w/o UV	100	95	97	92
#4 w/o UV	71	94	94	94
#5 w/o UV	88	90	88	85
#6 w/o UV	94	94	94	97
Mean	91	95	94	92
SD	11	3	3	4

	0 Time	10min	20min		30min
#1 UV irra	94	94		91	89
#2 UV irra	93	93		89	98
#3 UV irra	97	95		92	89
#4 UV irra	91	97		94	97
#5 UV irra	83	88		88	95
#6 UV irra	91	94		97	97
Mean	92	93		92	94
SD	5	3		3	4

Fig. 6

	Slow flow rate	
	w/o UV	UV irradiated
#1	13.67	13.28
#2	10.38	8.85
#3	9.31	9.31
#4	11.01	10.67
#5	10.6	12.15
#6	11.77	14.31
Mean	11.1	11.4
SD	1.5	2.2

	Fast flow rate	
	w/o UV	UV irradiated
#1	13.93	14.26
#2	9.38	16.92
#3	15.88	8.42
#4	7.88	8.55
#5	10.59	10.76
#6	16.85	16.6
Mean	12.4	12.6
SD	3.7	3.9

Fig. 7

	PLT count ($x10^6$/mL)	A265 (x100 diluted with PAS-3M)
#1	1.870	0.727
#2	1.120	0.630
#3	1.968	0.747
#4	2.200	0.685
#5	1.904	0.614
#6	1.980	0.636
#7	1.959	0.628
#8	1.960	0.625
#9	2.280	0.648
#10	1.937	0.694
#11	1.951	0.661
#12	0.795	0.473
#13	1.030	0.488
#14	1.190	0.555
#15	1.880	0.643
#16	1.980	0.632
#17	2.120	0.625
#18	1.980	0.637
#19	1.930	0.539
#20	1.611	0.545
#21	1.461	0.496
#22	1.978	0.603
#23	2.040	0.714
#24	2.280	0.766
#25	2.040	0.662
#26	2.090	0.678
#27	2.020	0.646

#28	1.201	0.622
#29	1.381	0.562
#30	1.975	0.643
#31	1.960	0.731
#32	1.991	0.610
#33	2.060	0.579
#34	1.947	0.622
#35	1.981	0.568
#36	1.973	0.617
#37	1.992	0.610
#38	2.040	0.604
#39	2.100	0.609
#40	0.896	0.488
#41	0.814	0.474
#42	2.120	0.604
#43	1.933	0.495
#44	1.967	0.602
#45	1.039	0.463
#46	1.141	0.493
#47	2.220	0.669
#48	0.982	0.443
#49	1.202	0.529
#50	1.925	0.602
#51	1.970	0.568
#52	2.120	0.642
#53	1.980	0.551
#54	1.980	0.674
#55	1.894	0.581
#56	2.100	0.514
#57	1.967	0.540
#58	1.963	0.599
#59	0.860	0.481
#60	1.158	0.512
#61	1.951	0.611
#62	2.080	0.660
#63	2.140	0.652
#64	1.972	0.687
#65	0.968	0.559
#66	1.213	0.593
#67	1.146	0.576
#68	2.040	0.671
#69	1.988	0.671
#70	1.004	0.494
#71	1.978	0.617
#72	2.020	0.670
#73	1.980	0.624
#74	1.886	0.619
#75	1.919	0.678
#76	1.081	0.487
#77	0.961	0.446
#78	0.995	0.446
#79	1.004	0.493
#80	0.878	0.491
#81	2.100	0.699
#82	1.117	0.500
#83	1.814	0.556
#84	2.140	0.601
#85	1.025	0.462
#86	1.947	0.656
#87	1.931	0.712
#88	1.123	0.457
#89	0.891	0.442
#90	0.990	0.521
#91	1.974	0.561
#92	1.999	0.552
#93	2.040	0.606
#94	0.998	0.774
#95	1.987	0.600
#96	2.220	0.704
#97	1.845	0.559
#98	1.343	0.475
#99	1.127	0.447
#100	1.277	0.528
#101	1.943	0.649
#102	2.020	0.586
#103	1.327	0.384
#104	1.197	0.465
#105	1.956	0.615
#106	2.100	0.662
#107	0.853	0.590
#108	1.044	0.493
#109	0.928	0.565
#110	1.015	0.572
#111	0.852	0.500
#112	1.213	0.574
#113	1.500	0.621
#114	2.160	0.679
#115	1.248	0.570
#116	1.226	0.559
#117	0.865	0.447
#118	2.060	0.675
#119	0.932	0.502
#120	1.993	0.572
#121	1.968	0.558
#122	1.078	0.510
#123	2.020	0.654
#124	1.980	0.623
#125	2.180	0.612
#126	1.990	0.558
#127	1.907	0.659
#128	1.975	0.578
#129	0.966	0.514
#130	2.060	0.727
#131	0.831	0.460
#132	2.020	0.667

#133	2.080	0.678
#134	1.979	0.661
#135	1.892	0.561
#136	1.977	0.663
#137	0.994	0.471
#138	2.120	0.690
#139	1.327	0.658
#140	1.408	0.589
#141	1.188	0.652
#142	1.010	0.596
#143	1.984	0.615
#144	1.402	0.488
#145	1.946	0.644
#146	1.128	0.586
#147	1.951	0.581
#148	2.000	0.622
#149	1.367	0.583
#150	1.099	0.667
#151	2.000	0.627
#152	2.080	0.641
#153	2.020	0.609
#154	0.921	0.515
#155	1.035	0.567
#156	1.120	0.450
#157	1.925	0.630
#158	1.990	0.567
#159	1.984	0.678
#160	1.020	0.473
#161	2.040	0.568
#162	2.080	0.661
#163	1.082	0.607
#164	1.230	0.563
#165	1.087	0.527
#166	1.995	0.714
#167	1.061	0.570
#168	0.988	0.448
#169	1.199	0.583
#170	1.249	0.523
#171	1.079	0.595
#172	2.040	0.761
#173	1.937	0.602
#174	1.948	0.679
#175	1.987	0.644
#176	1.808	0.620
#177	1.161	0.550
#178	2.240	0.686
#179	2.160	0.692
#180	1.922	0.696
#181	2.020	0.636
#182	1.230	0.558
#183	1.170	0.590
#184	1.125	0.585
#185	1.137	0.556
#186	1.959	0.658
#187	1.961	0.573
#188	1.976	0.717
#189	1.692	0.609
#190	1.566	0.579
#191	2.140	0.639
#192	1.932	0.662
#193	1.860	0.560
#194	1.960	0.691
#195	1.971	0.620
#196	1.080	0.578
#197	2.120	0.687
#198	2.260	0.674
#199	1.209	0.558
#200	1.053	0.548
#201	1.973	0.688
#202	2.100	0.596
#203	1.998	0.658
#204	1.195	0.614
#205	1.002	0.552
#206	2.100	0.751
#207	1.970	0.644
#208	2.220	0.728
#209	2.080	0.659
#210	1.980	0.693
#211	2.140	0.733
#212	0.820	0.611
#213	0.839	0.503
#214	2.000	0.674
#215	0.895	0.485
#216	2.020	0.627
#217	1.993	0.705
#218	2.100	0.688
#219	2.180	0.771
#220	2.000	0.687
#221	2.120	0.739
#222	1.993	0.720
#223	1.004	0.537
#224	1.994	0.726
#225	2.140	0.687
#226	2.100	0.749
#227	1.855	0.740
#228	0.807	0.473
#229	0.785	0.472
#230	2.080	0.675
#231	2.320	0.736
#232	0.897	0.502
#233	2.040	0.715
#234	2.020	0.655
#235	1.995	0.691
#236	2.120	0.759
#237	1.023	0.546

#238	2.140	0.700
#239	1.935	0.686
#240	1.045	0.547
#241	1.717	0.622
#242	1.957	0.621
#243	1.993	0.705
#244	1.007	0.553
#245	1.996	0.658
#246	1.869	0.639
#247	1.902	0.693
#248	1.954	0.741
#249	1.951	0.717
#250	1.108	0.604
#251	1.987	0.638
#252	0.872	0.521
#253	2.000	0.666
#254	1.023	0.570
#255	0.829	0.483
#256	1.988	0.692
#257	2.040	0.599
#258	1.919	0.675
#259	1.089	0.600
#260	2.040	0.679
#261	1.922	0.650
#262	1.414	0.783
#263	1.753	0.640
#264	1.213	0.554
#265	1.512	0.586
#266	2.000	0.602
#267	1.532	0.595
#268	1.332	0.597
#269	1.433	0.563
#270	1.450	0.508
#271	1.916	0.655
#272	2.040	0.659
#273	2.140	0.689
#274	2.020	0.697
#275	1.574	0.540
#276	1.158	0.580
#277	1.545	0.564
#278	0.867	0.520
#279	1.980	0.611
#280	0.963	0.534
#281	2.080	0.658
#282	1.493	0.540
#283	1.460	0.590
#284	1.966	0.655
#285	2.040	0.599
#286	1.456	0.607
#287	2.080	0.670
#288	1.922	0.625
#289	2.000	0.703
#290	1.662	0.583
#291	1.423	0.532
#292	1.948	0.687
#293	1.988	0.648
#294	1.940	0.605
#295	1.642	0.623
#296	1.496	0.582
#297	1.881	0.637
#298	1.473	0.515
#299	1.960	0.634
#300	2.080	0.596
#301	1.952	0.686
#302	0.921	0.470
#303	2.100	0.703
#304	1.462	0.605
#305	1.335	0.670
#306	0.988	0.500
#307	1.969	0.621
#308	1.422	0.585
#309	1.898	0.592
#310	2.120	0.598
#311	2.160	0.688
#312	2.000	0.669
#313	1.518	0.564
#314	1.943	0.645
#315	1.633	0.580
#316	2.280	0.752
#317	2.180	0.663
#318	2.020	0.698
#319	2.100	0.709
#320	1.947	0.646
#321	2.140	0.701
#322	1.172	0.604
#323	1.082	0.516
#324	1.342	0.634
#325	1.763	0.676
#326	1.492	0.526
#327	1.769	0.653
#328	1.168	0.493
#329	0.880	0.488
#330	1.033	0.531
#331	0.997	0.490
#332	1.032	0.511
#333	0.970	0.486
#334	1.047	0.511
#335	1.188	0.535
#336	1.137	0.540
#337	1.084	0.644
#338	1.036	0.517
#339	1.169	0.506
#340	1.292	0.519
#341	1.279	0.537
#342	1.053	0.473

#343	1.158	0.521
#344	1.187	0.525
#345	0.998	0.496
#346	1.050	0.500
#347	1.176	0.528
#348	0.978	0.530
#349	0.975	0.437
#350	1.131	0.509
#351	1.120	0.504
#352	1.072	0.484
#353	0.955	0.442
#354	0.920	0.488
#355	0.906	0.478
#356	1.073	0.497
#357	1.108	0.552
#358	1.061	0.470
#359	1.071	0.470
#360	0.894	0.465
#361	1.028	0.468
#362	1.103	0.598
#363	0.914	0.443
#364	1.181	0.598
#365	0.948	0.435
#366	1.030	0.499
#367	1.054	0.483
#368	1.046	0.525
#369	0.952	0.490
#370	1.054	0.566
#371	1.950	0.678
#372	1.078	0.491
#373	0.961	0.463
#374	1.964	0.641
#375	1.693	0.592
#376	1.098	0.592
#377	1.216	0.489
#378	0.914	0.457
#379	0.909	0.501
#380	1.288	0.581
#381	1.155	0.600
#382	1.190	0.641
#383	0.942	0.527
#384	0.992	0.537
#385	0.975	0.847
#386	1.053	0.473
#387	0.983	0.510
#388	1.210	0.538
#389	1.444	0.576
#390	1.154	0.527
#391	0.985	0.462
#392	1.648	0.655
#393	1.216	0.573
#394	1.238	0.568
#395	1.354	0.667
#396	1.137	0.498
#397	1.119	0.473
#398	1.393	0.545
#399	1.265	0.471
#400	1.859	0.637
#401	1.076	0.704
#402	1.986	0.562
#403	1.326	0.604
#404	1.754	0.558
#405	1.652	0.501
#406	1.069	0.581
#407	1.174	0.540
#408	1.323	0.552
#409	1.308	0.575
#410	1.496	0.562
#411	1.331	0.570
#412	1.317	0.554
#413	1.625	0.686
#414	1.322	0.606
#415	1.335	0.610
#416	1.333	0.618
#417	1.418	0.619
#418	1.140	0.663
#419	1.338	0.611
#420	1.178	0.554
#421	1.117	0.571
#422	1.103	0.546
#423	1.365	0.555
#424	1.472	0.528
#425	1.371	0.507
#426	1.305	0.561
#427	1.182	0.495
#428	0.992	0.468
#429	1.405	0.534
#430	1.856	0.620
#431	1.148	0.518
#432	1.300	0.541
#433	1.131	0.492
#434	1.274	0.532
#435	1.185	0.513
#436	1.409	0.560
#437	1.319	0.565
#438	1.767	0.640
#439	1.272	0.543
#440	1.101	0.649
#441	1.159	0.483
#442	1.215	0.547
#443	1.024	0.561
#444	1.703	0.575
#445	1.184	0.516
#446	1.356	0.551
#447	1.001	0.489

#448	1.397	0.592
#449	1.347	0.515
#450	1.341	0.578
#451	1.311	0.493
#452	1.344	0.526
#453	1.248	0.707
#454	1.242	0.529
#455	1.255	0.565
#456	1.283	0.518
#457	1.265	0.510
#458	1.223	0.587
#459	1.110	0.607
#460	1.585	0.530
#461	1.578	0.598
#462	1.576	0.590
#463	1.196	0.553
#464	1.076	0.496
#465	1.536	0.563
#466	1.141	0.514
#467	1.094	0.527
#468	0.978	0.525
#469	1.177	0.533
#470	0.945	0.515
#471	1.013	0.512
#472	1.164	0.469
#473	1.107	0.598
#474	1.150	0.520
#475	1.240	0.641
#476	1.033	0.579
#477	1.028	0.579
#478	1.008	0.503
#479	1.153	0.506
#480	1.109	0.524
#481	1.298	0.576
#482	1.001	0.531
#483	1.143	0.540
#484	1.736	0.682
#485	1.461	0.577
#486	1.010	0.515
#487	1.271	0.604
#488	1.107	0.534
#489	1.146	0.547
#490	1.226	0.578
#491	1.373	0.583
#492	1.402	0.651
#493	1.232	0.507
#494	1.227	0.570
#495	1.304	0.541
#496	1.251	0.563
#497	1.250	0.561
#498	1.222	0.619
#499	1.047	0.520
#500	1.570	0.608
#501	1.010	0.527
#502	1.064	0.563
#503	1.044	0.524
#504	1.373	0.635
#505	1.253	0.540
#506	1.292	0.547
#507	1.381	0.524
#508	1.231	0.616
#509	1.260	0.552
#510	1.215	0.590
#511	1.236	0.581
#512	0.894	0.523
#513	1.343	0.683
#514	1.085	0.603
#515	1.277	0.525
#516	1.325	0.572
#517	1.012	0.495
#518	1.352	0.607
#519	1.181	0.484
#520	1.130	0.595
mean	1.504	0.586
SD	0.439	0.077
CV	0.292	0.132
number	520	520
